# Efficient Photothermoelectric Conversion of CSS@BP/Bi_2_Te_3_ Array for Innovative Aircraft Attitude Recognition

**DOI:** 10.1002/advs.202414438

**Published:** 2025-03-11

**Authors:** Liangshutong Zhang, Yupu Zhang, Xinyu Li, Donghao Han, Wei Zhai, Jianyuan Wang

**Affiliations:** ^1^ MOE Key Laboratory of Materials Physics and Chemistry under Extraordinary Conditions & Shaanxi Provincial Key Laboratory of Condensed Matter Structure and Properties School of Physical Science and Technology Northwestern Polytechnical University Xi'an 710072 P. R. China

**Keywords:** aircraft attitude rrecognition, Bi_2_Te_3_, black phosphorus, copper sulfoselenide, photothermoelectric conversion

## Abstract

The realization of fast, simple and efficient flight attitude recognition is crucial for flight safety and control stability, but still faces challenges in new materials and technologies. Herein, a chloroplast‐like selenium‐doped copper sulfide@black phosphorus (CSS@BP) composite material is prepared by ultrasonic chemical synthesis using BP nanosheets to effectively absorb light energy and disperse CSS layers to promote rapid photothermal conversion, which shows the temperature change more than ≈40 °C and an excellent photothermal conversion efficiency of 68.9% at 405 nm, corresponding to the theoretical calculation results. Moreover, the CSS@BP/Bi_2_Te_3_ photothermoelectric conversion array prepared by pulsed laser deposition coated Bi_2_Te_3_ thermoelectric layer and laminated porous insulating polyimide film can generate rapid thermal current changes through irradiated/non‐irradiated thermal gradients. Hence, a portable attitude recognition box (ARB) is assembled with a based CSS@BP/Bi_2_Te_3_ array with a self‐balancing laser and a current measurement chip that enables accurate attitude recognition through the bidirectional current generated by changes of irradiated area. Excitably, the ARB demonstrates over 86.47% accuracy without complex algorithms, showing excellent stability and robustness. Thus, this work offers an innovative solution for advancing photothermal materials and low‐cost high‐precision flight attitude sensing technologies.

## Graphical abstract

A chloroplast‐like CSS@BP composite with superior photothermoelectric conversion performance is synthesized via sonochemical methods. A portable Attitude Recognition Box (ARB) incorporating a CSS@BP/Bi_2_Te_3_ photothermal conversion array is developed, which achieves precise attitude recognition through bidirectional current detection induced by irradiated area variations, showcasing high stability.

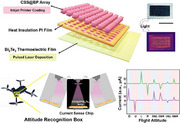

## 1 Introduction

In recent years, various types of aircraft, especially a variety of micro‐unmanned aerial vehicles, have been widely used in military, medical, rescue, photography, and other fields, playing a more significant role in society.^[^ ^1^ ^]^ In practical applications, the accurate identification of aircraft attitude is very important to the flight safety of aircraft. Previous reports have shown that establishing a coordinate system centered on the aircraft itself and accurately measuring its attitude (level flight, pitch, roll, yaw angles, etc.) is the key to ensuring the stable flight of the aircraft along the predetermined trajectory.^[^ ^2^, ^3^, ^4^, ^5^, ^6^, ^7^ ^]^ Effective attitude control can prevent unstable flight, position deviation, and accidental maneuvering of the aircraft. Moreover, the aircraft control system (e.g., autopilot) relies on accurate attitude data to adjust the flight control surface and propulsion system in real‐time. In addition, engineers and technicians can provide effective flight diagnosis and periodic maintenance analysis by monitoring and recording the precise attitude data of the aircraft. Additionally, when executing precision outdoor missions, it is particularly important to ensure that the aircraft's flight attitude remains within a controlled range for successful mission completion.

Several traditional attitude recognition techniques have been proposed, such as Kalman filtering, which fuses data from multiple sensors (e.g., Inertial measurement units (IMUs), Global Positioning System, visual sensors) to estimate flight attitude.^[^ ^8^, ^9^, ^10^ ^]^ In addition, visual‐inertial navigation utilizes cameras or other visual sensors to identify feature points through image processing algorithms,^[^ ^11^, ^12^, ^13^ ^]^ and combines with IMU data for attitude estimation. Another approach involves constructing a multilayer perceptual model to learn and process sensor signals to obtain attitude estimates.^[^ ^14^, ^15^ ^]^ The neural network method has also been used for this purpose.^[^ ^16^ ^]^ However, the limited robustness and adaptability of the measurement systems, the necessity for specific sensor placement locations, the high precision requirements for sensor probes, and the significant signal noise that can easily compromise the accuracy and reliability are still serious constraints for the development of aircraft attitude recognition technology. Moreover, most of the currently proposed methods require complex algorithms, making it challenging to accurately estimate aircraft attitude in a short time, while the relatively large volume also greatly increases production costs for small aircraft, hindering their widespread adoption. Therefore, improving the stability and environmental adaptability of sensors, while further simplifying the data processing process and increasing the portability of the device to achieve convenient, fast, and accurate aircraft attitude recognition, is a prominent problem that needs to be solved urgently.

To address this challenge, developing efficient and cost‐effective attitude recognition techniques for aircraft represents an effective research solution. The creation of lightweight and portable composite materials with targeted functionalities forms the foundation and key to these advancements. In recent years, 2D materials have garnered significant attention for their potential to enhance the performance of sensing materials, owing to their unique structural and functional properties. Black phosphorus (BP) is a novel 2D material with numerous unique physicochemical properties, among which its excellent photothermal conversion property has garnered significant attention.^[^ ^17^, ^18^, ^19^ ^]^ This attracted us to explore the feasibility of utilizing this advantage of BP's photothermal performance through new material composite technology and applied it to the development of new flight attitude control sensing materials. The adjustable band gap of BP allows it to efficiently absorb energy from sunlight or other light sources. Upon photon absorption, BP converts this energy into heat, releasing light absorption in a non‐radiative manner. The light absorption of BP predominantly spans the wavelength range from 400 to 2500 nm, within which BP can effectively convert light energy into heat. Thanks to the adjustable band gap of BP, which enables it to effectively absorb light across a wide range of wavelength spans, and its 2D structure ensures its exceptionally high photothermal conversion efficiency on the nanometer scale. Because of its excellent photothermal conversion performance, BP has been extensively used in diverse fields such as photothermal therapy, infrared imaging, photocatalysis, and environmental remediation. However, the limited environmental stability of BP has hindered its development. Researchers have attempted to enhance BP's stability and photothermal conversion performance by combining it with other materials, such as carbon nanotubes, metal nanoparticles, or other 2D materials.^[^ ^20^, ^21^, ^22^, ^23^ ^]^ Additionally, a novel multistage porous aerogel, prepared via solution blending of BP nanosheets, polyvinyl alcohol (PVA), and carbonized kapok (CK) fibers, demonstrated a life‐cycle safe multifunctional nanocomposite with properties of photothermal‐healing, thermal stability, and fire safety.^[^ ^24^ ^]^ Through the composite of a variety of materials, the stability of BP materials can be effectively improved, and if we can find a material with inherent photothermal effects to compound with BP, while improving the structural stability of BP materials, we can also use the synergistic effect between different components to enhance overall photothermal conversion efficiency.

Among many potential materials, we found that copper‐based materials have the potential to optimize BP performance, due to the discovery that BP is an inherent copper ion nanotrap. Hu et al. reported that the intrinsic Cu^2+^‐BP trapping ability can accelerate the degradation of photothermite while simultaneously enhancing photothermal stability.^[^ ^25^ ^]^ Copper sulfide (CuS) belongs to the copper‐based materials and has attracted our attention because of its stable 2D structure and excellent photothermal properties,^[^ ^26^, ^27^ ^]^ which is an ideal material with the potential for a compound with BP. However, the band gap of CuS is narrow, which restricts its light absorption band. Previous research work has demonstrated that its band gap can be adjusted by optimizing the synthesis method, doping, or altering its crystal structure, providing great operability for the improvement of its photothermal conversion performance. Among them, ternary Cu‐S‐Se semiconductor nanostructures,^[^ ^28^, ^29^, ^30^ ^]^ synthesized by changing the composition of sulfur compounds (i.e., S/Se ratio), have been proven to be an effective and practical approach for achieving tunable bandgap properties and enhancing light absorption at specific wavelengths. Xu et al. reported a simple, low‐temperature, controllable synthesis of ternary copper‐selenium alloys,^[^ ^31^ ^]^ which showcased continuous redshift in both the bandgap absorption and the near‐infrared localized surface plasmon resonance, indicating optical tuning capability. In addition, the composite exhibited superior electrocatalytic activity, with a 135% increase in power conversion efficiency (PCE) compared to the noble metal platinum counter electrode. Barman et al. effectively synthesized CuSe, CuS, and Cu‐S‐Se via chemical deposition methods and examined the impact of varying Se/S composition ratios on the optical and electrical properties of these systems. Additionally, they explored the potential applicability of Cu‐S‐Se in energy conversion devices. The above research indicates that it is effective to construct composite structures with improved performance by Se doping of CuS. However, so far, the combination selenium‐doped copper sulfide and BP to enhance the overall photothermal properties and stability of the material has not been explored.

Many green plants in nature can effectively absorb and quickly use light energy, which is inseparable from their chloroplast structure. In this work, the chloroplast‐like selenium‐doped copper sulfide@black phosphorus (CSS@BP) composite material was successfully prepared by ultrasonic chemical synthesis using selenium‐doped copper sulfide (CuS_y_Se_1‐y_, 0 ≤ y ≤ 1, hereafter referred to as CSS) and BP as raw materials by imitating the microstructure and light absorption function of chloroplasts. The CSS@BP composite structure uses the thin and large BP nanosheets like thylakoid frames as a skeleton to efficiently absorb light, while the CSS like chlorophyll as small lamellar clusters distributed in BP layers to promote rapid photothermal energy conversion. The CSS@BP composites exhibit excellent photothermal performance. The photothermal conversion efficiency of CSS@BP reaches 68.9% under 405 nm illumination, showing excellent light absorption characteristics, and has noticeable advantages in relevant research. The established photothermal conversion model explains that the significant performance enhancement is mainly due to carrier migration at the BP‐CSS interface, promoting the localized Surface Plasmon Resonance (LSPR) effect within the CSS, while the suppression of radiative relaxation within BP drives the non‐radiative relaxation process, further enhancing the light energy to heat conversion, and is augmented by phonon coupling effects.

On this basis, an innovative CSS@BP/Bi_2_Te_3_ photothermoelectric conversion array was successfully prepared by using Bi_2_Te_3_ with excellent thermoelectric properties to compound with CSS@BP. Owing to the outstanding photothermal conversion capability of the CSS@BP composite, a feature critical for sensors as it not only amplifies the temperature signal detected by the bottom Bi_2_Te_3_ thermoelectric layer but also improves efficiency and aids in further energy conservation, the array rapidly identifies the angle of incident light via the thermal currents induced by the temperature differential between the illuminated and non‐illuminated areas. Further, a portable attitude recognition box (ARB) was assembled based on the CSS@BP/Bi_2_Te_3_ array. Based on the relative angle changes between the incident light and the array under different flight attitudes, the ARB can achieve the real‐time accurate measurement of the roll and pitch angle through the change of the bidirectional current value of the array in different light receiving area, with an accuracy level of more than 86.47%. The ARB's black box and tiny volume design permit easy attachment to any part of a small aircraft without compromising its functionality, providing robust and stable performance that is unaffected by weather and environment. The ARB based on CSS@BP/Bi_2_Te_3_ array can be integrated with the Global Positioning System, Beidou Navigation Satellite System or other tools to realize real‐time estimation of air aircraft roll angle, pitch angle, and yaw angle of the aircraft. This work provides an innovative approach for the structural design of photothermoelectric conversion materials and the development of high‐precision aircraft attitude recognition technology.

## 2 Results and Discussion

### 2.1 Preparation and Application of the CSS@BP/Bi_2_Te_3_ Photothermoelectric Conversion Array

**Figure 1:**
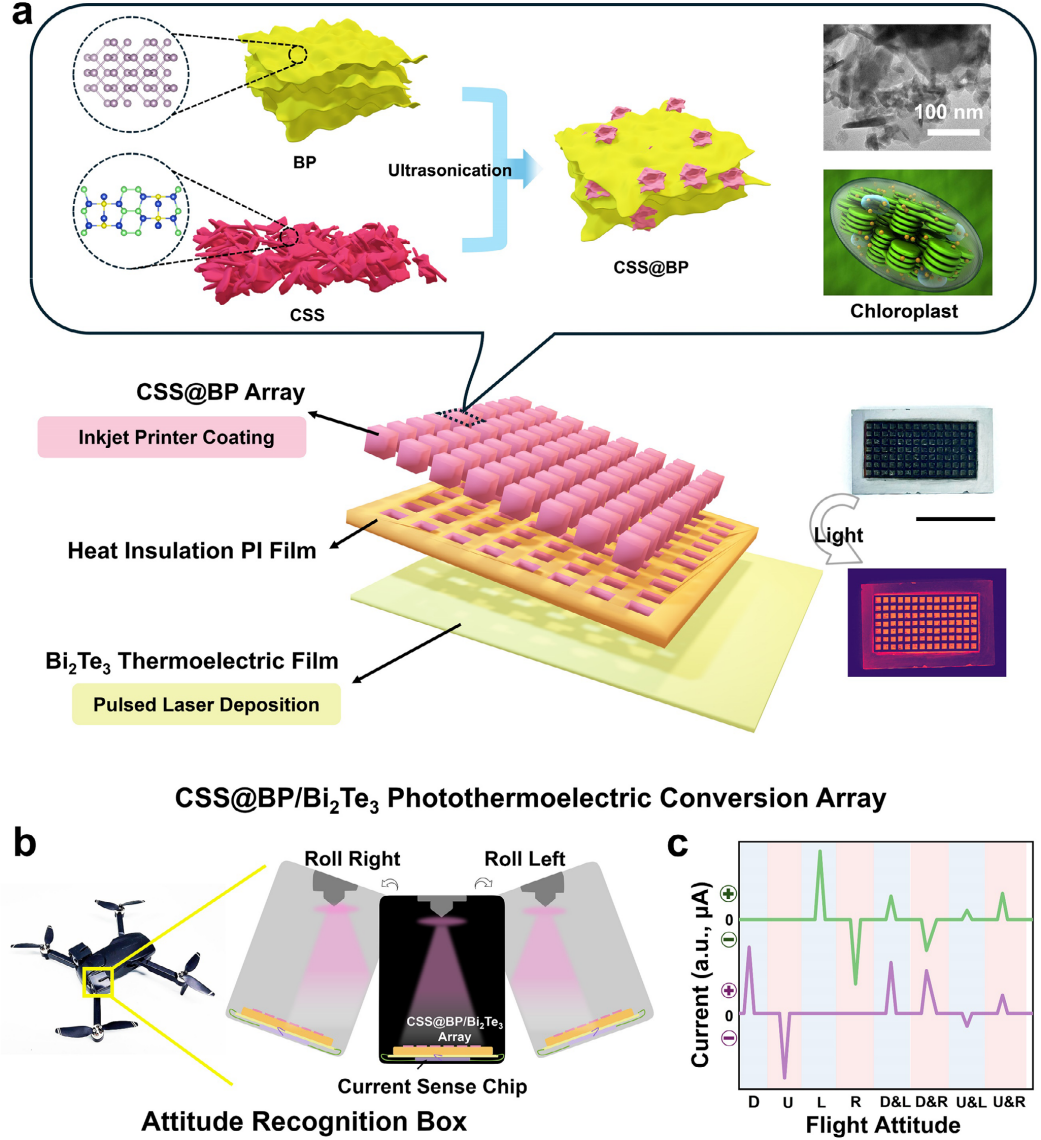
CSS@BP/Bi_2_Te_3_ photothermoelectric conversion array. a) Schematic and physical representation of the array structure; b) Exterior view of the attitude‐recognition box on a drone and a perspective view of its interior application; and c) Current values at different flight attitudes.

The structure of the CSS@BP/Bi_2_Te_3_ photothermoelectric conversion array is shown in **Figure** **1**a, which mainly consists of three components: the CSS@BP composite array, the PI film with regular hole patterns, and the Bi_2_Te_3_ thermoelectric film. The above illustration of Figure 1a illustrates the preparation process of the CSS@BP composite with a strong chemical crosslinked structure, using fewer layers of BP and optimized CSS (see the Experimental Section for details) by ultrasonic chemical synthesis. The scanning electron microscope (SEM) image of CSS@BP composite and the chloroplast structural details are shown on the right side of the illustration. In detail, the obtained CSS@BP composite takes full advantage of the large, thin lamellar structure of BP and the small, ordered lamellar stack of CSS, in which BP serves as the structural framework with CSS interspersed and layered within, analogous to the organization of the thylakoid frame and chlorophyll in chloroplasts.^[^ ^32^ ^]^ This configuration effectively enhances light absorption and the material's rapid energy conversion capabilities.

First, the CSS@BP composite as the top layer is uniformly deposited on the PI film layer using an inkjet printer. At the same time, the PI film has been laser‐engraved to create a regularly patterned hole structure, taking advantage of the excellent thermal insulation properties of the PI film to ensure that the heat generated by the photothermal effect of each array unit remains isolated from adjacent units during light exposure. Consequently, when exposed to light, infrared thermography reveals significant warming only in the individual CSS@BP array unit, while the other areas show essentially no temperature variation, as demonstrated in the right inset of Figure 1a. In addition, the underlying Bi_2_Te_3_ thermoelectric film prepared by pulsed laser deposition (refer to Figure S1, Supporting Information) exhibits superior thermoelectric properties. It is capable of generating directional currents driven by temperature gradients, thereby providing an indication of temperature changes within the system. When the direction of the light source is fixed, variations in the array's deflection alter the exposed surface area and specific locations, resulting in changes to the temperature generated by each unit. Consequently, temperature differences arise within the array's surface, which are reflected in the thermal current produced by the Bi_2_Te_3_ thermoelectric film, where there is a one‐to‐one correspondence exists between the current's direction and magnitude and the angle of deflection. Based on this principle, we have developed the ARB shown in Figure 1b. The exterior of the ARB is a light‐proof black box that ensures complete isolation from external light sources. Inside the box, a miniature laser light source, integrated with a compact gyroscope, is securely mounted on the top. This setup guarantees that the light source is always emitted perpendicular to the ground. At the lower end of the box, the electrodes are attached to each of the four edges of the CSS@BP/Bi_2_Te_3_ photothermoelectric conversion array (with opposing edges sharing the same color, namely a purple electrode pair and a green electrode pair). These electrodes are connected via wires to a current detection chip located on the base, enabling real‐time monitoring of the current flowing through both electrode pairs. When the aircraft is in a horizontal position, meaning there is no pitching or rolling, the laser light is uniformly distributed across the array, resulting in a uniform photothermal effect over the entire surface. Consequently, there is no temperature difference across the surface, and the currents measured by both the violet and green electrodes are zero. When the aircraft pitches downward, no temperature difference is observed in the direction of the green electrodes, so no current is generated there. However, the violet electrodes experience a positive current, which is recorded as “violet positive, green zero.” Conversely, when the aircraft pitches upward, the display indicates “violet negative, green zero.” In the case of a leftward tilt, no current is generated in the direction of the violet electrodes, while the green electrodes produce a positive current, resulting in a display of “violet zero, green positive.” Similarly, during a rightward roll, no current is observed in the direction of the green electrodes, leading to a display of “violet zero, green negative.” Additionally, various scenarios such as downward dive combined with a leftward roll, downward dive combined with a rightward roll, upward elevation combined with a leftward roll, and upward elevation combined with a rightward roll are illustrated in Figure 1c. This figure details the changes in the currents of the violet and green electrodes for different orientations. By analyzing the specific magnitudes of these currents, the corresponding deflection angles can be deduced, so as to accurately reflect the aircraft's flight attitudes. This method provides an innovative and straightforward approach for determining the aircraft's flying orientation.

### 2.2 Preparation and Characterization of CSS@BP Composites

The primary raw materials for CSS@BP composites are pure CSS and BP. First, the optimal selenization ratio of copper sulfide was systematically investigated (details can be found in the Supporting Information). A series of CuS_y_Se_1‐y_ (where y ranges from 0 to 1) compositions with varying S/Se ratios were synthesized via a hydrothermal method. By systematically measuring and comparing the temperature increase under illumination, a distinct correlation between the S/Se ratio and photothermal conversion efficiency was identified. Among these samples, the composition exhibiting the highest photothermal conversion temperature across different wavelengths was determined to be y = 0.5 (CuS_0.5_Se_0.5_). This specific composition, henceforth abbreviated as CSS, was subsequently selected as the primary subject for further intensive investigation.

**Figure 2:**
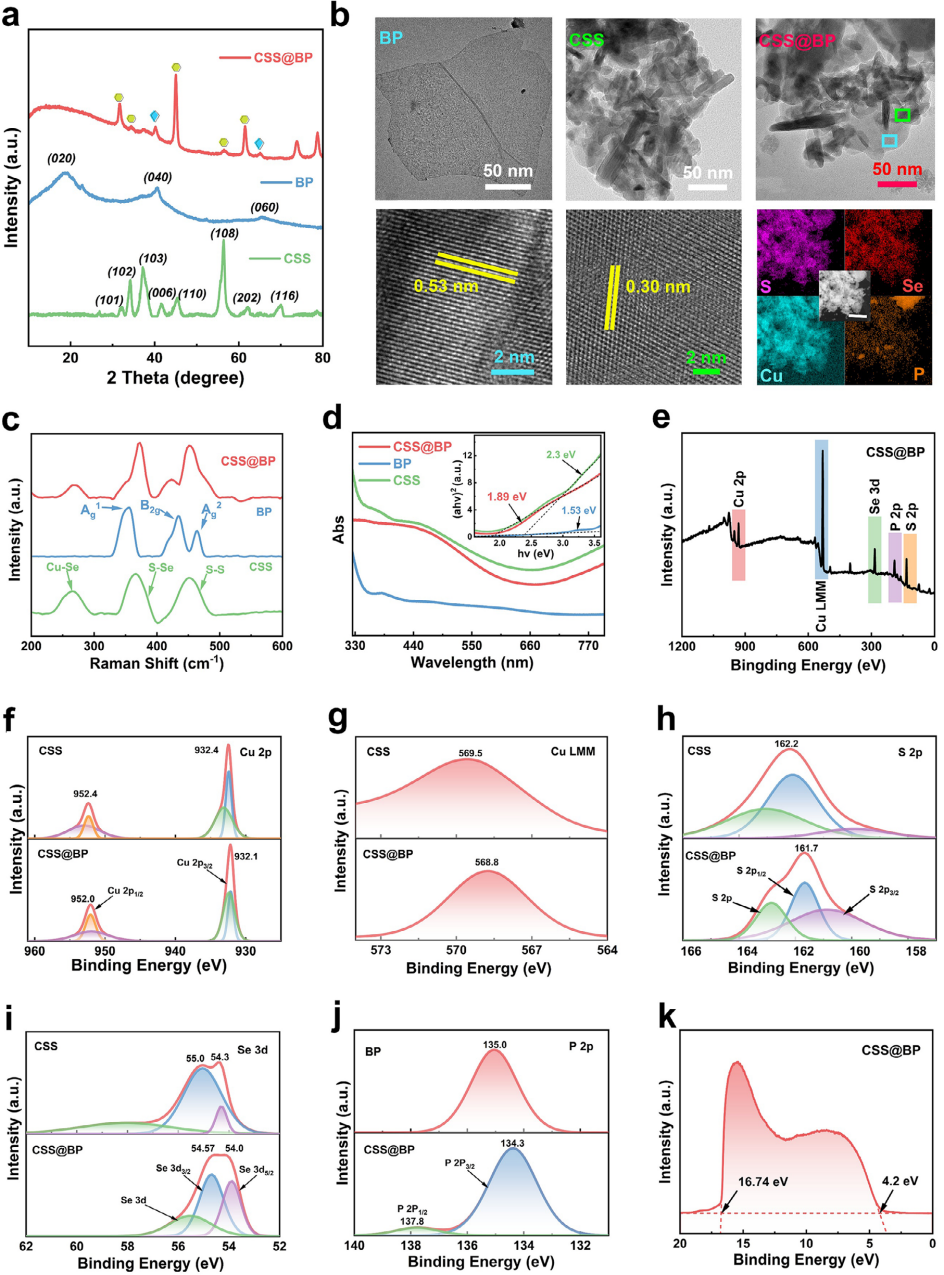
Characterization of CSS@BP composites. a) XRD patterns of CSS, BP, and CSS@BP; b) TEM images (top row) and HR‐TEM images of BP, CSS, and CSS@BP, along with EDS energy scans; c) Raman spectra of the intrinsic materials and the composites; d) UV–vis spectra of the three materials, with the inset showing the computed band gap; e) XPS wide‐scan spectrum of CSS@BP; comparison of f) Cu 2p, g) Cu LMM, h) S 2p, i) Se 3d, and j) P 2p high‐resolution spectra of the intrinsic CSS and CSS@BP; k) UPS spectra of CSS@BP composites.

Regarding BP, bulk BP powder was first synthesized from red phosphorus using a high‐energy ball milling method. BP nanosheets were then successfully prepared through an ultrasound‐assisted liquid‐phase exfoliation method.The CSS@BP composites were obtained by ultrasonically co‐mixing the BP nanosheets and CSS powder in anhydrous ethanol for 3 h. As shown in **Figure** **2**a, the physical phase of CSS powder aligns with the hexagonal Cu phase criterion, with its diffraction peaks corresponding to the (101), (102), (103), (110), (108), and (116) planes of Cu. Additionally, the diffraction peaks at 27°, 32°, 45.3°, and 56.4° reflect the advantageous hexagonal phases of CuSe and CuS, as well as the less favorable cubic phase of CuSe. The BP nanosheets exhibit characteristic peaks (020), (040), and (060) at 18.9°, 40.5°, and 65.5°, corresponding to their lamellar structure. The X‐Ray diffraction (XRD) patterns of the CSS@BP composites exhibit the characteristic peaks of both CSS and BP. Specifically, the peaks at 31.84°, 34°, 45.3°, 56.4°, and 62.1° correspond to CSS, while the (040) and (060) diffraction planes of BP are also evident. The peak positions have been marked using icons that correspond to the colors of the patterns for both materials, as illustrated in the accompanying figures. Overall, the XRD patterns confirm the successful synthesis of the composites. It is noteworthy that the polycrystalline nature of the BP nanosheets results in their (020)‐faceted characteristic peaks appearing as gently sloping peaks in the composites. The observed change in peak intensity following the composite process is primarily attributed to lattice rearrangement and selective orientation induced by ultrasound. Additionally, the slight shift in the positions of the diffraction peaks suggests an interaction between the two components, rather than a mere physical mixing. Figure 2b shows the transmission electron microscope (TEM) image of BP, revealing a large, thin 2D layered structure with good transmittance and sizes up to ≈200 nm. In contrast, the TEM image of CSS, shown on the right, depicts a relatively small, thick lamellar structure that often exhibits regular pentagonal or hexagonal shapes. The CSS nanosheets range in size from tens to about one hundred nanometers and tend to agglomerate due to their small size, resulting in a pronounced lining when observed under a transmission electron microscope. After ultrasonic treatment, the CSS powder was fully dispersed and adhered to large BP nanosheets, which acted as a backbone, as depicted in the rightmost section of the first row in Figure 2b. This resulted in a noticeable reduction in the agglomeration of the composite material observed under TEM. High‐resolution TEM (HRTEM) analysis revealed that the lattice spacing of the composite BP was 0.53 nm, while that of CSS was 0.30 nm, indicating only minor changes relative to the intrinsic materials. The distribution of various elements in the composite was more clearly observed through energy dispersive spectroscopy (EDS). Elements S and Se were found to be present in equal amounts and distributed in numerous small clusters throughout the material, similar to the Cu elements. The BP nanosheets, which serve as the backbone, are uniformly distributed in large areas and their content is relatively low compared to the other elements, consistent with observations in the TEM images of the composite. To further determine the composition of the composites, Raman spectroscopy was conducted. As shown in Figure 2c, the two peaks at 265.4 cm−¹ and 366.5 cm−¹ for the intrinsic CSS material correspond to the Se‐Se stretching mode and the S‐Se stretching mode of the S‐Se ions, respectively, while the peak at 454 cm−¹ is attributed to the S‐S stretching mode. Conversely, BP exhibits three peaks at 354.5, 435.7, and 462.5 cm−¹, which correspond to the three phonon vibrational modes unique to BP: A_g_^1^, B_2_ _g_, and A_g_^2^. The significant presence of these Raman modes in the spectra of the composites confirms the successful preparation of the composite material. Additionally, the weaker vibrational intensity of BP compared to CSS aligns with the fact that BP, serving as a backbone, is present in only small amounts necessary to support the overall structure. Ultraviolet‐visible (UV–vis) absorption spectroscopy was also utilized to analyze the composition, content, and structure of the materials. As illustrated in Figure 2d, the absorption properties of the composite material fall between those of the two intrinsic materials, with a notably enhanced absorption peak in the 440–500 nm range. The absorption spectrum allows for the estimation of the material's band gap, which, after calculation, is found to be 1.89 eV. This band gap value is intermediate between that of BP (1.53 eV) and CSS (2.3 eV), indicating that the band gap engineering has been effectively adjusted, allowing precise control over the composition and structure of the composite material. This results in the material demonstrating a significant advantage in absorption within the 400–500 nm range. Additionally, the chemical properties of the material surface were characterized using X‐ray photoelectron spectroscopy (XPS). The XPS wide spectrum (Figure 2e) reveals strong signals for the elements Cu, S, Se, and P, confirming the elemental composition of the composite. A further comparison of the elemental high‐resolution spectra of the intrinsic material and the CSS@BP composite reveals that the Cu 2p orbital electrons exhibit two peaks at ≈952.0 and 932.0 eV, as shown in Figure 2f, indicating the presence of divalent Cu^2+^. The positions of these peaks remain largely unchanged before and after the composite formation. This stability is attributed to the challenge XPS faces in distinguishing the chemical state of the Cu element. To further elucidate the chemical state changes, we employed Auger electron spectroscopy to measure the LMM spectrum of Cu (Figure 2g), which offers greater differentiation. The LMM spectrum shows a single peak that shifts from 569.5 to 568.8 eV before and after composite formation. This shift suggests that the Cu element predominantly remains in the Cu^2+^ valence state throughout the system, with the peak shift to a lower energy state and increased image symmetry indicating that Cu atoms tend to gain electrons during the compositing process. In Figure 2h, the S 2p spectrum of CSS shows two peaks at 163.0 and 162.0 eV corresponding to S 2p and S 2p_1/2_, respectively. In contrast, the peak at 160.0 eV corresponds to S 2p_1/2_, with S‐elemental p orbital electrons predominantly in the lower energy state. In CSS@BP, the peak positions remain similar, but the peak intensities across different valence states tend to equalize, suggesting an increase in electron migration to higher energy states and a higher likelihood of energy exchange with the environment. The Se 3d spectrum of CSS (Figure 2i) displays three distinct photoelectron peaks at binding energies of 58.1, 55.0, and 54.3 eV, corresponding to Se 3d, Se 3d_3/2_, and Se 3d_5/2_, respectively. In CSS@BP, however, the Se d‐orbital electrons migrate to lower energy states during the compositing process, resulting in energy loss and a change in electron spin direction. Finally, Figure 2j shows that after compositing, the P 2p spectrum reveals two peaks at 137.8 and 134.3 eV, corresponding to P 2p_1/2_ and P 2p_3/2_, respectively. The UV photoelectron spectra (UPS) of CSS@BP shown in Figure 2k provide additional evidence regarding the changes in the surface valence of the composites. According to the following formula,^[^ ^33^ ^]^

(1) $$\phi_{\;sam}=hv-\left|{E_{k}}^{cutoff}-{E_{k}}^{Fermi}\right|$$

CSS (Figure S22, Supporting Information), BP (Figure S23, Supporting Information), and CSS@BP, the work functions are calculated to be 6.15, 7.62, and 8.68 eV, respectively. The work function of the composite material is significantly higher than that of the two intrinsic materials. This larger work function indicates that the composite surface has a greater capacity to bind electrons, making it less likely for non‐equilibrium carriers generated on the material's surface to be transported outward when photons are injected. This reduced likelihood of carrier transport inhibits energy dissipation and promotes the conversion of photon energy into thermal energy through phonon scattering, thereby enhancing the material's photothermal performance.

### 2.3 Photothermal Conversion Performance and Mechanism of CSS@BP Composites

**Figure 3:**
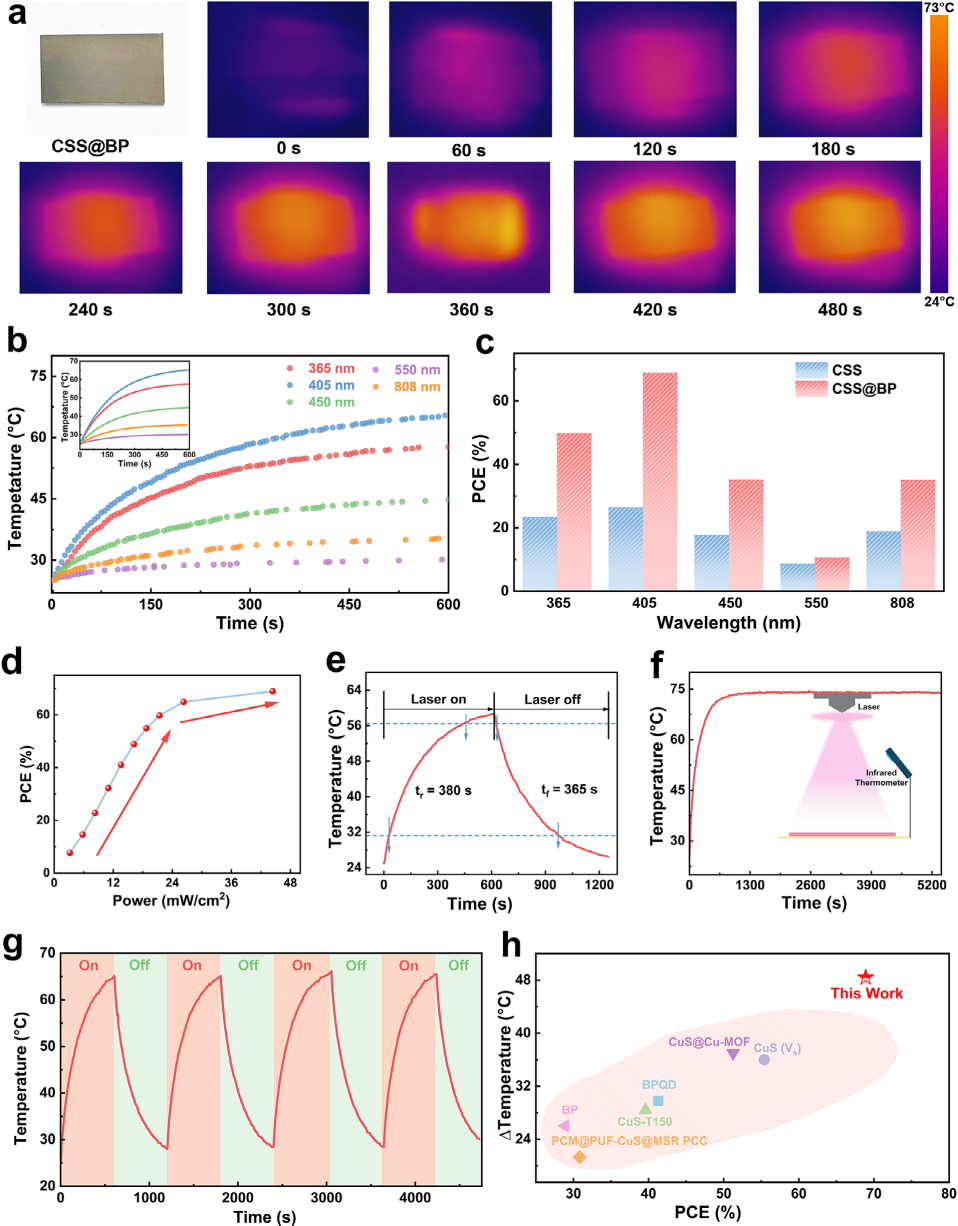
Photothermal performance of CSS@BP composites. a) Thermograms of CSS@BP at various time points under 405 nm light; b) Variation in surface temperature of the material over time under different wavelengths of light; c) Comparison of photothermal conversion efficiency between CSS and CSS@BP at various wavelengths of light; d) Variation in photothermal conversion efficiency with light intensity; e) Photothermal response time under 405 nm light; f) Temperature change during continuous light exposure; g) Temperature variation of the material during alternating light periods; h) Performance comparison with other similar studies.^[^ ^36^, ^37^, ^38^, ^39^, ^40^, ^41^, ^42^ ^]^

We further evaluated the photothermal conversion capability of CSS@BP composites under light illumination. **Figure** **3**a illustrates the temperature change on the surface of the composite material, as observed through infrared thermography under 405 nm wavelength light. CSS@BP dispersion was uniformly sprayed onto a glass substrate using an inkjet printer, vacuum dried, and subsequently irradiated with a 405 nm wavelength laser. Nearly immediately, the surface temperature of the sample began to rise, maintaining a rapid warming rate for 4 min before slowing down and eventually stabilizing at a constant temperature. Upon comparing the temperature increase due to photothermal effects under different light wavelengths (Figure 3b), we observed that CSS@BP exhibits the most exceptional photothermal performance under 405 nm wavelength light. This wavelength not only achieves the highest warming rate but also stabilizes at a maximum temperature of 72.4 °C, with a temperature increase of 48.4 °C from the initial temperature. This performance significantly surpasses that of pure CSS, indicating a superior photothermal conversion efficiency. To quantify the photothermal material performance more accurately, the photothermal conversion efficiency (η) is a crucial parameter. Here, we use the following equation^[^ ^34^ ^]^ to calculate the photothermal conversion efficiency η:

(2) $$\eta =\frac{hs\left(T_{max}-T_{sur}\right)-Q_{dis}}{I\left(1-10^{-a}\lambda \right)}$$

where *h* is the thermal conductivity, *s* is the surface area of the material, *T_max_* refers to the maximum temperature that the system can achieve through photothermal conversion under light, *T_sur_* denotes the ambient temperature and *Q_dis_* refers to the heat loss to the environment, *I* represent the intensity of the incident light, *a* is the absorbance of the material, and *λ* is the wavelength of the incident light. Our calculations reveal that the CSS@BP composites demonstrate significantly higher photothermal conversion efficiency compared to CSS across all bands (Figure 3c). Notably, in the 405 nm band, the photothermal conversion efficiency reaches 68.9%, indicating that the CSS@BP composites substantially enhance the photothermal conversion capability of the materials. We employed 405 nm LEDs for irradiation at different powers. It is worth noting that when switching the light source to different power settings, we had to cool the sample down to room temperature and allow an additional thermal relaxation time of 150 s to ensure that it fully reverted to its original state. Figure 3d shows the conversion efficiency exhibits an increasing trend with higher incident light intensity, thus, adjusting the incident light intensity during practical applications can improve the conversion efficiency and yield better results. The response time to reach 90% of the stabilized temperature is ≈380 s (Figure 3e). As shown in Figure 3f, the CSS@BP composites maintain the highest photothermal conversion temperature under continuous light exposure, demonstrating excellent long‐term stability. Additionally, during cyclic light switching tests, the material consistently exhibits a stable rising and falling trend, highlighting its outstanding stability (Figure 3g). The photothermal conversion capability and stability of CSS@BP composites also show superior performance compared to other similar studies, as illustrated in Figure 3h.

**Figure 4:**
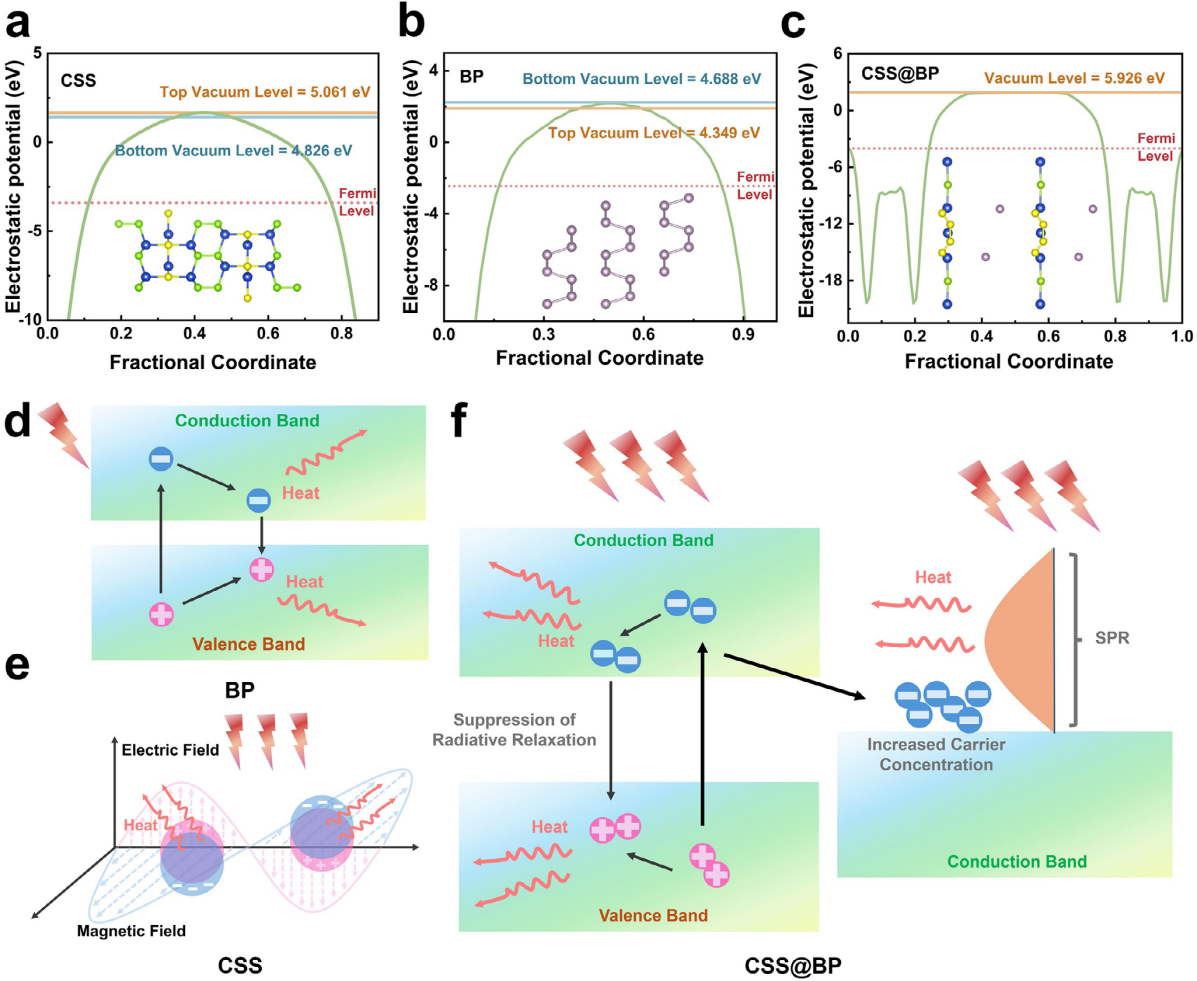
Mechanism of the photothermal effect in CSS@BP composites. Work functions of a) CSS, b) BP, and c) CSS@BP composites, the inset shows the models of all three; d) Mechanism of the photothermal response in intrinsic BP materials; e) Mechanism of the photothermal response in CSS materials; f) Internal mechanism underlying the enhanced photothermal conversion capacity of CSS@BP composites.

To investigate the photothermal conversion mechanism of CSS@BP composites, we conducted simulations employing first‐principles calculations. The work functions of CSS (refer to **Figure** **4**a), BP (refer to Figure 4b), and CSS@BP composites (refer to Figure 4c) were computed independently. It is widely recognized that the work function of a semiconductor corresponds to the difference between its Fermi level and vacuum energy level. Our calculations indicate that CSS@BP composites exhibit a work function of up to 5.926 eV, surpassing that of pure CSS and BP. The work function quantifies the energy necessary to dislodge an electron from a solid. When photon energy is imparted to excite an electron, the electron must surmount the potential barrier at the solid's surface plane to successfully detach. A higher work function in the composite material signifies a notably enhanced ability to bind electrons, thereby making electrons less readily excitable, and leading the light energy to be less likely dissipated through electrical energy conversion. We propose that the outstanding photothermal conversion capability of CSS@BP composites arises from the synergistic enhancement and functional complementarity of CSS and BP. As illustrated in Figure 4d, the photothermal effect in BP is primarily attributed to its internal nonradiative relaxation. BP's light absorption largely depends on its intrinsic absorption band gap. Upon light excitation, photon energy injection causes valence‐band electrons to jump to the conduction band, generating electron‐hole pairs. The energy of the excited electrons can be dissipated in two ways: first, by photon scattering, and second, through nonradiative relaxation of phonons, which transfers the energy to the lattice. This nonradiative relaxation induces local lattice vibrations, leading to an overall increase in temperature and a macroscopic conversion of light energy into heat. The efficiency of this conversion process in BP is strongly influenced by the material's light‐absorbing properties and surface characteristics. In contrast, the photothermal conversion effect of CSS, a defect‐structured semiconductor, is mainly attributed to localized surface plasmon resonance^[^ ^35^ ^]^ (LSPR), as shown in Figure 4e. This phenomenon can be attributed to the migration of carriers on the surface, akin to the effect caused by defects, which generates a localized surface plasmon resonance (LSPR) effect on the nanoparticles similar to that observed in metals. Defects induced by doping lead to substantial surface carrier migration, and coherent oscillations occur when the vibrational frequency of the incident light approaches the resonance frequency of the free electrons, resulting in collective excitation of the electrons. The excited hot electrons resonate with the incident electromagnetic field, potentially leading to some electrons undergoing a transition and contributing to an electric current, while others emit electromagnetic waves. This process transfers energy in the form of phonon‐phonon coupling, radiating heat and causing a temperature increase. The plasmonic resonance effect of CSS is directly related to the concentration of surface carriers and is largely independent of nanoparticle morphology, which imparts superior photostability. The CSS@BP composite enhances surface carrier migration, with S and P elements losing some of their electrons. Photon energy injection excites the valence electrons in BP, generating nonequilibrium carriers that migrate toward the CSS side at the interface, thereby promoting the LSPR effect of CSS. Additionally, based on the test results from UV‐vis and UPS, we observe that the work function of the composite material has experienced a substantial enhancement compared to the two intrinsic materials. The composite material's capability to strongly bind electrons on its surface allows it to further suppress the radiative relaxation of electrons. Consequently, light energy is more likely to be released in the form of non‐radiative relaxation, which facilitates the conversion of light energy into heat energy. Furthermore, potential phonon coupling between BP and CSS also contributes positively to the photothermal conversion process. Collectively, these mechanisms ensure the efficient absorption and conversion of light energy into thermal energy in CSS@BP composites (Figure 4f).

### 2.4 Application and Testing of CSS@BP/Bi_2_Te_3_ Photothermoelectric Conversion Arrays

**Figure 5:**
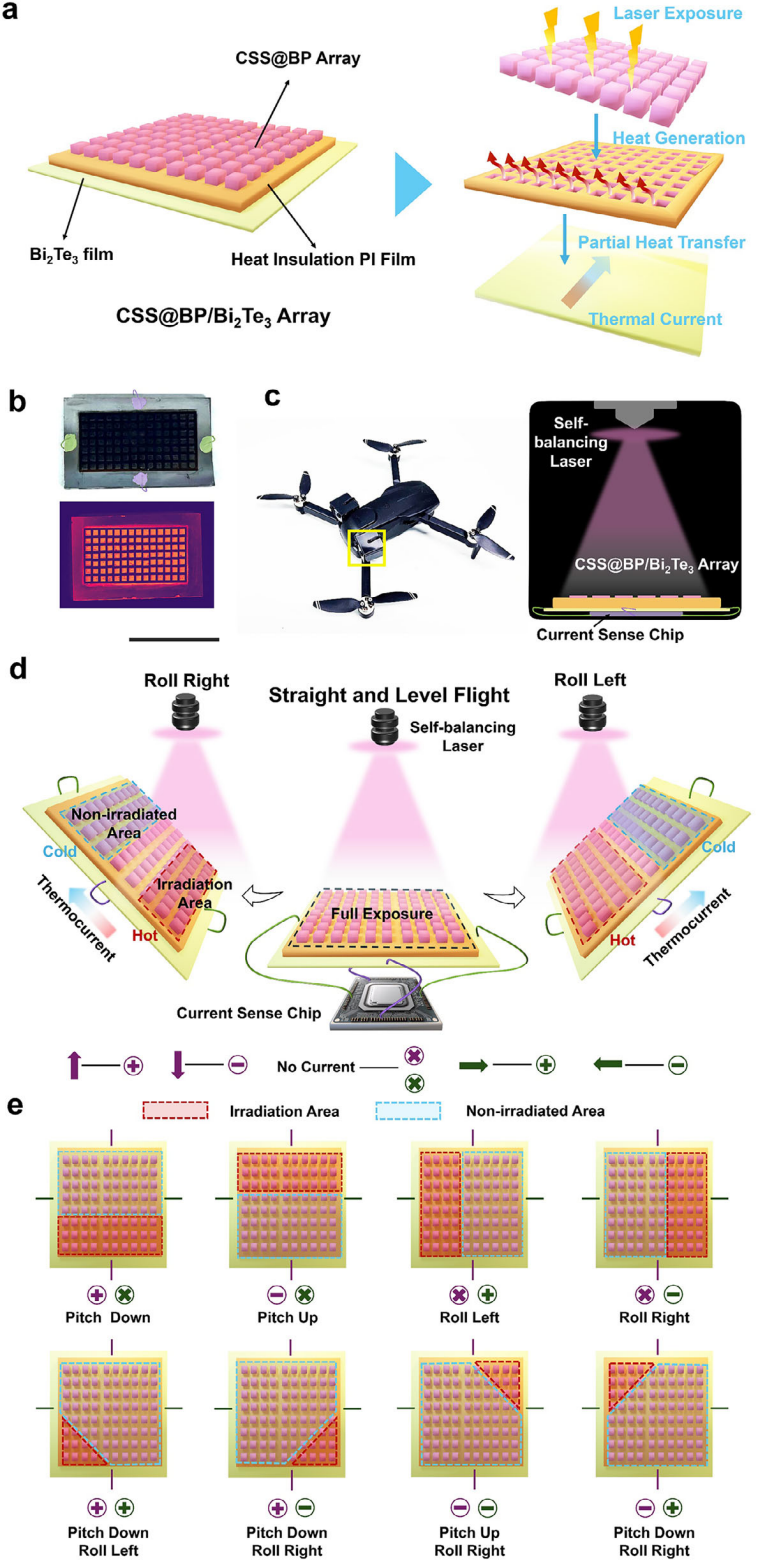
Application of the CSS@BP/Bi_2_Te_3_ photothermal conversion array. a) Schematic diagram of the array structure and the photothermal conversion process; b) Physical diagram of the array and the thermal image under light illumination; c) Physical diagram of the ARB with the integrated photothermal heat conversion array and its internal structure; d) Mechanism of internal photothermoelectric conversion during aircraft deflection; e) Polarity of violet‐green electrodes in relation to light state and flight attitude.

We developed a CSS@BP/Bi_2_Te_3_ photothermoelectric conversion array by first applying CSS@BP composites, known for their exceptional photothermal conversion properties, onto the PI film with patterned hole arrays using an inkjet printer. This was followed by transferring the coated PI films onto a Bi_2_Te_3_ thermoelectric film deposited via pulsed laser deposition, as illustrated in **Figure** **5**a. When a portion of the cells is exposed to laser irradiation, the CSS@BP in those cells experiences a temperature increase due to the photothermal effect. In contrast, the cells not exposed to the laser maintain room temperature owing to the excellent insulating properties of the PI film. This creates a localized temperature gradient on the surface of the system, which induces a thermoelectric effect in the thermoelectric thin film, generating currents that flow from the hot region to the cold region. Consequently, the varying sizes of the irradiated regions result in changes in the thermocurrent values, as shown in Figure 5b. As depicted in Figure 5c, we constructed a 7 × 14 photothermoelectric conversion array featuring two pairs of electrodes, green and violet, positioned on opposite sides. When the laser irradiates different areas of the array, the violet and green electrodes produce distinct signals due to the photothermoelectric conversion mechanism. The polarity and magnitude of these signals can be used to accurately determine the location of the laser irradiation. Utilizing this array, we fabricated a small aircraft attitude recognition box (ARB). The ARB features a fully opaque black exterior. Inside, the CSS@BP/Bi_2_Te_3_ photothermoelectric conversion array is mounted at the bottom and connected to a current measurement chip via two pairs of electrodes, colored purple and green, to facilitate real‐time monitoring of two separate current outputs. A laser, integrated with a miniature gyroscope, is positioned on the top of the ARB. When the ARB is placed flat, the laser uniformly covers the surface of the array. Any tilting of the ARB causes the photothermoelectric conversion array, fixed at the bottom, to tilt accordingly. However, due to the gyroscope's stabilization, the laser remains perpendicular to the ground. This results in an angle between the incident light and the array. Following the conversion process from light to heat to electricity, a current is generated across the violet and green electrodes.

When the aircraft is in a smooth flight state, the incident light fully covers the photo‐thermoelectric conversion array, resulting in no temperature differential on the surface, and both the violet and green electrode currents are zero, placing them at the origin of the coordinate axis. When the aircraft's front end maintains a zero‐pitch angle but begins to roll to the right (i.e., pitch angle = 0, roll angle ≠ 0), the incident light shifts to the lower right region, as illustrated in Figure 5d (left). Consequently, the right part of the photo‐thermoelectric array begins to warm up, creating a hot zone, while the upper left part, which does not receive light, retains room temperature, forming a cold zone. This results in a thermal current in the green electrode, flowing from the hot region to the cold region, while the violet electrode does not produce a current due to the absence of a temperature difference. The situation reverses when the air aircraft rolls to the left. In the field of aerial aircraft, flight attitude is typically described using the roll angle and pitch angle. The roll angle refers to the rotation of the aircraft around its longitudinal axis (front to rear), while the pitch angle refers to the rotation around its lateral axis (left to right). The green electrode is associated with the roll angle, and the violet electrode is associated with the pitch angle. By combining the positive and negative values of the two sets of electrodes, the eight possible flight attitudes of the aircraft can be described, as shown in **Figure** **5**e. The currents corresponding to four of these attitudes (pitch down, pitch up, roll left, and roll right) are described as outlined above. When both pitch and roll angles are nonzero, such as in a pitch down and roll left situation, the violet electrode experiences a temperature gradient with a hot zone in the lower part and a cold zone in the upper part, resulting in an upward current. Similarly, the green electrode encounters a hot zone on the left and a cold zone on the right, generating a rightward current. In the right‐angled coordinate system. Analogous conclusions can be drawn for other flight attitude directions.

**Figure 6:**
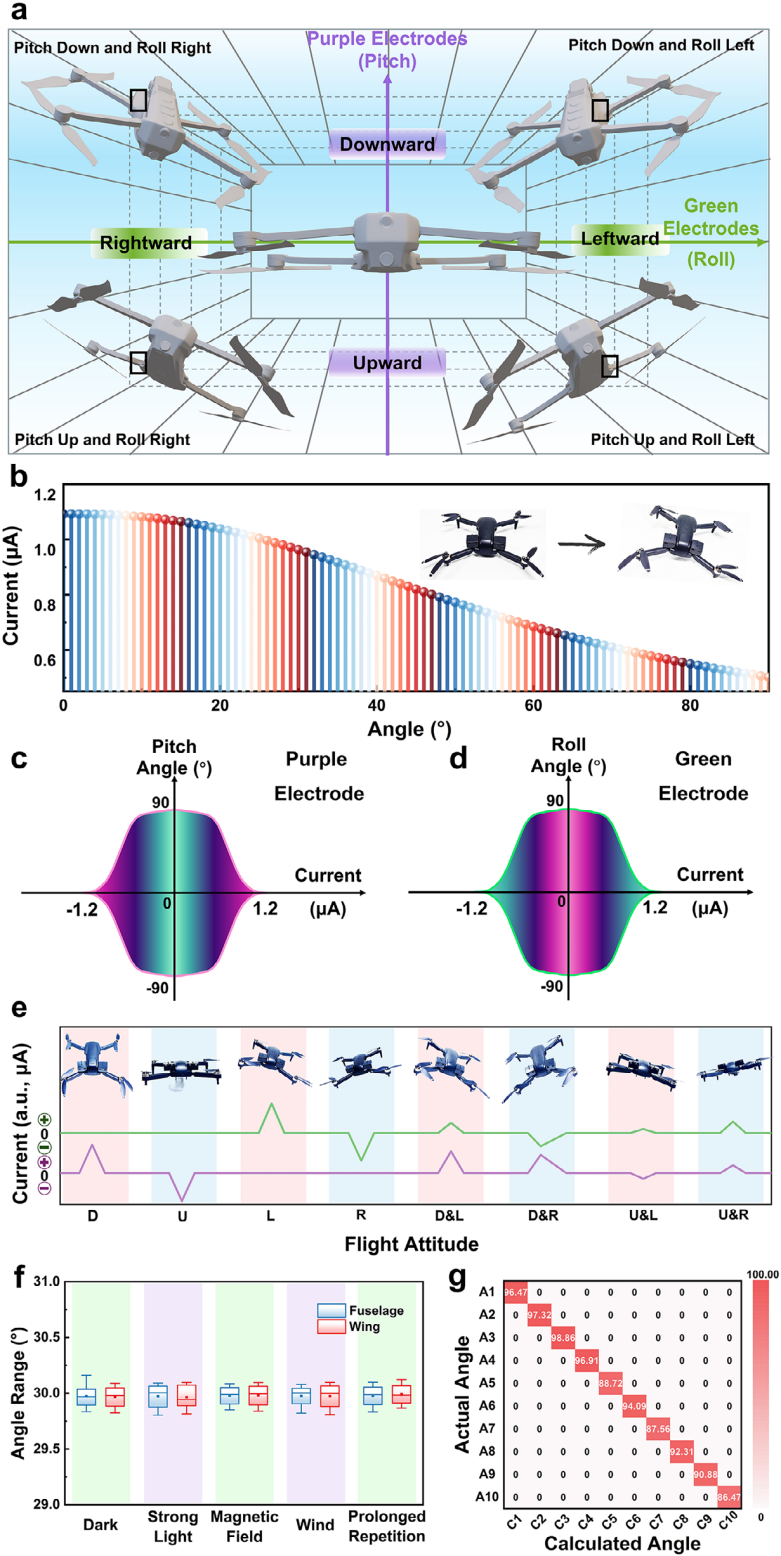
Practical application tests of ARB. a) Correspondence diagrams of different flight attitudes with the violet‐green electrode current indications; b) Variation in current value with the angle of deflection when deflected in a single direction; c,d) Direction of current and number of indications of violet and green electrodes at different changes of angle; e) Violet and green current indications of ARB in eight different motion states; f) Measurements of ARB at fuselage and wing in different states; g) Confusion matrix for ARB measurement of flight attitude.

The relationship between the aircraft's attitude in space and the currents from the violet and green electrodes can be approximated using the right‐angle coordinate axes depicted in **Figure** **6**a. In this coordinate system, the horizontal axis represents the current indications from the green electrode, and the vertical axis represents the current indications from the violet electrode. Positive current in the green electrode corresponds to a leftward tilt, while negative current corresponds to a rightward tilt. Positive current in the violet electrode indicates a downward tilt and negative current indicates an upward tilt. When the violet and green electrodes are in the first quadrant, the aircraft is in a pitch‐down and roll‐left state. In the second quadrant, the air aircraft is in a pitch‐down and roll‐right state. The third quadrant corresponds to a pitch‐up and roll‐right state, and the fourth quadrant indicates a pitch‐up and roll‐left state.

After initially identifying the type of flight attitude based on the direction of the currents, it is essential to establish a correlation between the current values and the tilt angles to determine precise pitch and roll angle measurements. To achieve this, we conducted experiments by rolling the small aircraft equipped with ARB at various angles and measuring the corresponding current values on the green electrodes, as depicted in Figure 6b. Our observations revealed that as the angle increased, the current value rose rapidly at first and then more slowly. By fitting multiple sets of data, we derived a relationship between the current and the deflection angle using the formula:

(3) $$A=a_{0}\left[1+exp\left(x-a_{1}\right)\right]-a_{2}$$

where a_0_, a_1_, and a_2_ are constants without direct practical significance in this context.

Their specific values are provided in the Supplementary Information. This formula enables us to accurately estimate the pitch and roll angles based on the measured current values. As a result, a one‐to‐one correspondence can be established between the current readings of the violet and green electrodes and the flight attitude. Figure 6c,d illustrates the current indications of the violet and green electrodes across various states, where color shades symbolize angle variations. The direction and magnitude of the currents from the violet and green electrodes correspond to Pitch and Roll, respectively, thereby enabling unique determination of the roll angle's type and orientation.

In 3D space, with the vehicle serving as the axis, the vehicle's flight attitude can be categorized into eight types: Pitch Down, Pitch Up, Roll Left, Roll Right, Pitch Down Roll Left, Pitch Down Roll Right, Pitch Up Roll Left, and Pitch Up Roll Right. We assessed the currents in the violet‐green electrodes across each of the eight states, as presented in Figure 6e, and scrutinized the two sets of current indications, which were found to exhibit a one‐to‐one correspondence with the flight attitude. Consequently, in practical application, our ARB operates as outlined in Figure S24 (Supporting Information). Upon a change in the vehicle's attitude, induces alterations in the irradiation area. The ensuing temperature differential drives the currents in the violet and green electrodes, wherein the direction of the violet electrode current denotes the direction of the pitch angle, and its magnitude signifies the deflection angle of the pitch angle. Conversely, the direction of the green electrode current indicates the direction of the roll angle, and its value corresponds to the deflection angle of the roll angle. Ultimately, this enables the precise measurement of the vehicle's eight motion attitudes. This measurement can be conducted in a remarkably fast state. Following testing, we observed that the device is capable of responding to angle changes with a response time of 0.17 s, demonstrating good sensitivity (as shown in Figure S25, Supporting Information). Indeed, the key to the ARB's exceptional performance lies in the design of the close contact between the CSS@BP and the thermoelectric film, coupled with effective thermal isolation between each cell in the array. This configuration ensures that the photothermoelectric array can respond clearly and sensitively to changes in angle, enabling rapid and accurate detection of angular variations. The combination of these design elements significantly enhances the ARB's sensitivity and reliability, making it a highly effective solution for attitude recognition in various applications. The robustness of the ARB is of significant concern to us. As illustrated in Figure 6f, the boxes denote the upper and lower interquartile ranges, the central horizontal line signifies the mean value, and the whiskers correspond to the maximum and minimum values of the test data. Our tests demonstrate that the estimated angle error of the ARB, whether mounted on the fuselage or the wing, does not exceed 0.2° across various environments (the specific environmental parameters are shown in Table S2, Supporting Information). To further assess its stability, we conducted repeated successive measurements by varying the attitude every 1 min over a period of 30 min. The results indicate that the drift of the estimated angle of the ARB is minimal, with the error remaining consistently below 0.15°. These findings underscore the outstanding stability of the ARB. As shown in Figure 6g, we randomly selected ten true angular values within the range of 0° to 90°. For each of these ten deflection angles, we generated 30 sets of predictions. Subsequently, we compared each set of predictions in the dataset with their corresponding actual flight attitude angles. The results of these tests indicate that the probability of the error between the predicted and actual values being within 0.05° is greater than 86.47%. This finding validates the high accuracy and reliability of the state identification and mapping method based on photocurrents. Moreover, in Table S3 (Supporting Information), ^[^ ^43^, ^44^, ^45^, ^46^, ^47^ ^]^ we present a comparison of the absolute error between this study and other relevant research. This comparison serves to underscore the superiority of our proposed approach in terms of sensing accuracy.

Furthermore, we can also utilize spherical ARBs to achieve finer measurements by distributing the photothermoelectric converter arrays throughout the interior, thereby accommodating extreme application scenarios such as 360° flipping. This recognition method offers notable simplicity. In general, visual sensor estimation methods are limited by environmental conditions such as nighttime and bright light scenarios. Magnetometer‐based methods are susceptible to magnetic field interferences, while gyroscope methods may experience measurement errors under strong wind conditions. Furthermore, advanced computational techniques like Kalman filtering and machine learning require high‐precision hardware, which can increase costs significantly. In contrast, the ARB benefits from the overall airtight design, making it virtually immune to environmental factors such as sunlight, wind speed, and magnetic fields. The integrated design also contributes to its compact size, allowing it to be mounted on any part of the aircraft and adaptable to various flight environments and installation scenarios. The ARB stands out due to its small size, lightweight, and cost‐effectiveness, which can lead to significant cost savings and holds promise for integration into small unmanned aerial vehicles (UAVs). Although its accuracy may not match the current state‐of‐the‐art technologies, it offers substantial cost savings and has the potential for wide application in civil UAVs. In high‐precision flight applications, it can be integrated with other identification systems to provide reliable references and corrections for attitude estimation.

## 3 Conclusion

In this study, CSS@BP composites were successfully prepared by ultrasonically assisting the attachment of Se‐doped CuS (i.e., CSS) to the surface of BP using large‐sized BP nanosheets as the backbone. The tests demonstrated that these composites exhibit exceptional photo‐thermal conversion capabilities, significantly outperforming the intrinsic materials. Under laser irradiation, the composites achieved a maximum temperature increase of 48.4 °C, with a photo‐thermal conversion efficiency reaching 68.9%. Furthermore, the photothermal conversion demonstrated by these composites is highly reproducible and stable over time, which represents a considerable advantage compared to similar studies. We analyze that the photothermal effect of BP eigenmaterials primarily originates from the phonon nonradiative relaxation of semiconductor valence electrons triggered by photoexcitation. Conversely, CSS eigenmaterials achieve photothermal conversion predominantly through the Localized Surface Plasmon Resonance (LSPR) effect of surface electrons. The CSS@BP composites significantly enhance the system's photothermal conversion capability by facilitating the generation of nonequilibrium carriers and suppressing radiative relaxation. Additionally, phonon coupling contributes to this enhancement.

Leveraging the photothermal properties of CSS@BP composites, we have developed a CSS@BP/Bi_2_Te_3_ photothermoelectric conversion array. This array comprises CSS@BP arrays, a PI insulating film, and a Bi_2_Te_3_ thermoelectric film. When a portion of the array is illuminated, the exposed unit experiences warming due to the photothermal effect, while the unexposed unit maintains ambient temperature. The resulting temperature difference across the surface induces current generation in the thermoelectric film. The direction of the current and the number of oscillations can be utilized to indicate the angle between the array and the incident light. Further, we have developed an aircraft attitude recognition box (ARB) based on a CSS@BP/Bi_2_Te_3_ photothermoelectric converter array. The self‐balancing laser positioned on top of the ARB maintains vertical irradiation, while the photothermoelectric converter array at the bottom shifts in response to changes in the aircraft's attitude. By measuring the current values from two sets of electrodes fixed on all four surfaces, the aircraft's flight attitude can be determined in real‐time. With an accuracy exceeding 86.47% and an absolute error within 0.2°, the ARB facilitates rapid and dependable identification. This novel attitude recognition method is characterized by its simplicity in data processing, rapid analysis capabilities, and absence of the need for additional sensors or complex image recognition algorithms, presenting a new and feasible approach to aircraft attitude recognition.

## 4 Experimental Section

### Materials

The Bismuth telluride target (25 × 3 mm, 99.99%) was procured from Deyang Aijialun New Materials Co. The polyimide (PI) film with a square hole array (thickness 0.7 mm) was supplied by Tianjin Huanoprice Technology Co. Anhydrous ethanol (C_2_H_5_OH, ≥ 99.8%, AR) was purchased from Tianjin Fuyu Fine Chemical Co. Red phosphorus (RP, ≥ 99.99%) and selenium powder (Se, ≥ 99.999%) were obtained from Aladdin Chemical Co. Sodium hydroxide (NaOH, AR) was sourced from Guangdong Guanghua Technology Co. Copper nitrate trihydrate (Cu(NO_3_)_2_·3H_2_O, AR) and precipitated sulfur (S, ≥ 99.5%) were purchased from Sinopharm Chemical Reagent Co.

### Preparation of CuS_y_Se_1‐y_

Sulfur powder and selenium powder were dissolved in varying proportions in 20 mL of 5.0 m NaOH aqueous solution, heated to 80 °C, and stirred for 30 min. Then, add 5 mL of 0.2 m Cu(NO_3_)_2_ ethanol solution was added, and the mixture was thoroughly, mixed before being transferred to a hydrothermal reactor. The reactor was heated at 100 °C for 10 h. After the reaction, the product was washed thoroughly with deionized water and anhydrous ethanol and then dried to obtain CuSySe1‐y powder.

### Preparation of Black Phosphorus Nanosheets

Inside an argon glove box, red phosphorus powder was mixed with an appropriate amount of large and small steel balls in a ball milling jar. The jar was sealed and placed in a ball mill for 12 h to obtain black phosphorus powder. A portion of 400 mg of the black phosphorus powder was then added to 40 mL of anhydrous ethanol and stirred for 4 h to ensure full dispersion. The solution was subsequently sonicated in an ice water bath for 10 h. The solution was centrifuged at 5000 rpm for 30 min, and the supernatant was collected as the ethanol dispersion of black phosphorus nanosheets.

### Preparation of CSS@BP Composite Material

30 mL of the black phosphorus nanosheet ethanol dispersion was taken, and 200 mg of CSS powder was added. The mixture was ultrasonicated in an ice water bath for 3 h. Using an inkjet printer within a glove box, the obtained solution was sprayed uniformly onto a glass substrate. After drying, the CSS@BP composite film is obtained.

### Preparation of Bi_2_Te_3_ Film

The high‐transmittance glass substrate was cleaned with alcohol and deionized water in multiple cycles, then dried and fixed on a heated sample stage within the pulsed laser deposition system. The system was evacuated to a stable pressure of 1 × 10^−4^ Pa. The substrate temperature was raised to 350 °C, and the substrate was positioned ≈10 cm from the Bi_2_Te_3_ target. The Bi_2_Te_3_ target was irradiated with a pulsed laser at a frequency of 10 Hz for 30 min to deposit the Bi2Te3 film onto the substrate.

### Preparation of CSS@BP Composite Photothermoelectric Conversion Arrays

A polyimide (PI) film with a square array of holes was affixed to the glass substrate on which the Bi_2_Te_3_ film had been deposited. The CSS@BP composite material was applied again using an inkjet printer in a glove box. After drying, the device was covered with a layer of highly transparent, ultra‐thin PVC film.

### Equipment

The surface morphology was characterized using a Field Emission Scanning Electron Microscope (FEI, Verios G4) and a Transmission Electron Microscope (FEI, Talos F200X). The phases of the material were characterized using an X‐ray Diffractometer (D8 DISCOVER A25). Raman spectra were obtained using a Micro Confocal Raman Spectrometer (WItec, Alpha300R), and absorption spectra were obtained using a UV–vis Spectrophotometer (Shimadzu, UV‐3600).

## Conflict of Interest

The authors declare no conflict of interest.

## Supporting information



Supporting Information

## Data Availability

The data that support the findings of this study are available from the corresponding author upon reasonable request.
