# Supplementary material for: Efficient Photothermoelectric Conversion of CSS@BP/Bi2Te3 Array for Innovative Aircraft Attitude Recognition
Source: Adv Sci (Weinh). 2025 Mar 11;12(17):2414438. doi: 10.1002/advs.202414438 (PMC12061242; doi:10.1002/advs.202414438)
Supplement: Supplementary file 1 — Supporting Information [file ADVS-12-2414438-s001.docx]

Supporting Information

Efficient Photothermoelectric Conversion of CSS@BP/Bi_2_Te_3_ Array for Innovative Aircraft Attitude Recognition

Liangshutong Zhang, Yupu Zhang*, Xinyu Li, Donghao Han, Wei Zhai and Jianyuan Wang*

L. S. T. Zhang, Y. P. Zhang, X. Y. Li, D. H. Han, W. Zhai and J. Y. Wang

MOE Key Laboratory of Materials Physics and Chemistry under Extraordinary Conditions & Shaanxi Provincial Key Laboratory of Condensed Matter Structure and Properties, School of Physical Science and Technology, Northwestern Polytechnical University, Xi’an 710072, P. R. China

E-mail: zhangyupu@nwpu.edu.cn; wangjy@nwpu.edu.cn


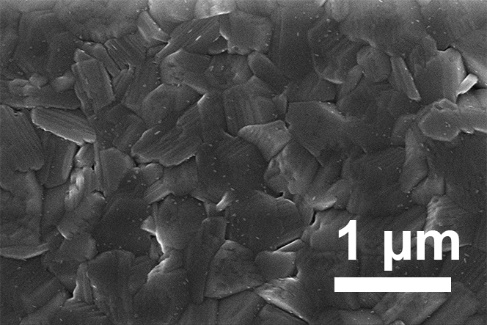


**Figure S1** SEM image of Bi_2_Te_3_ thin film.


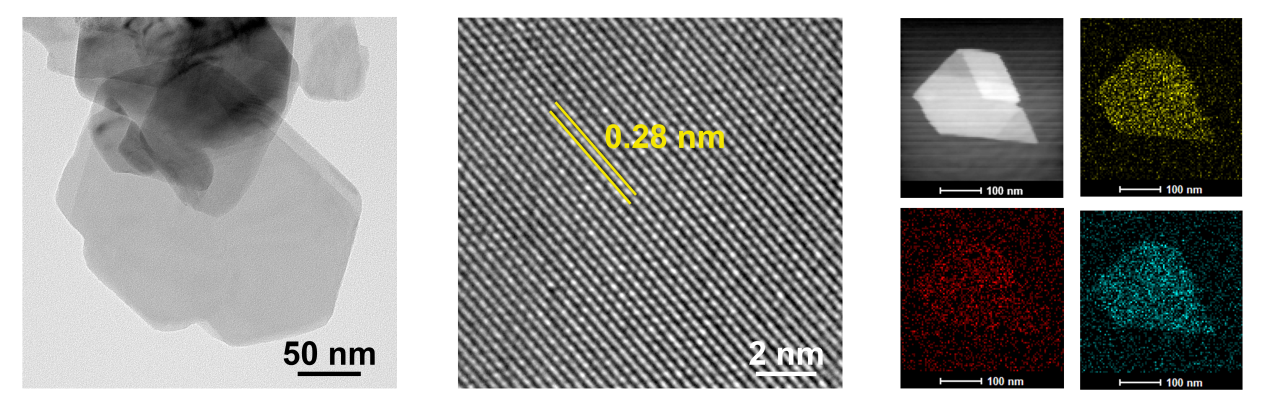


**Figure S2** TEM image (left), HRTEM image (center) and EDS energy spectrum (right) of CuS.


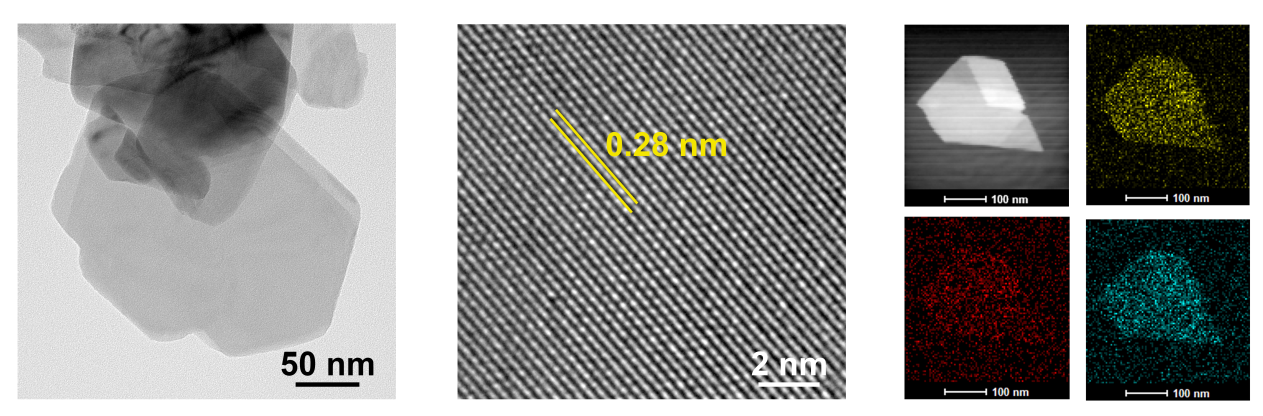


**Figure S3** TEM image (left), HRTEM image (center) and EDS energy spectrum (right) of CuS_0.6_Se_0.4_.


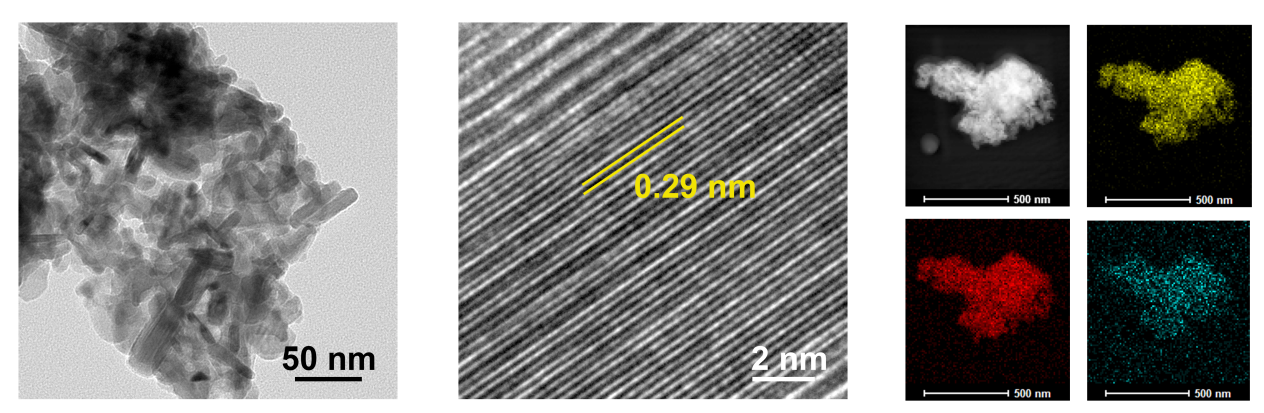


**Figure S4** TEM image (left), HRTEM image (center) and EDS energy spectrum (right) of CuS_0.5_Se_0.5_.


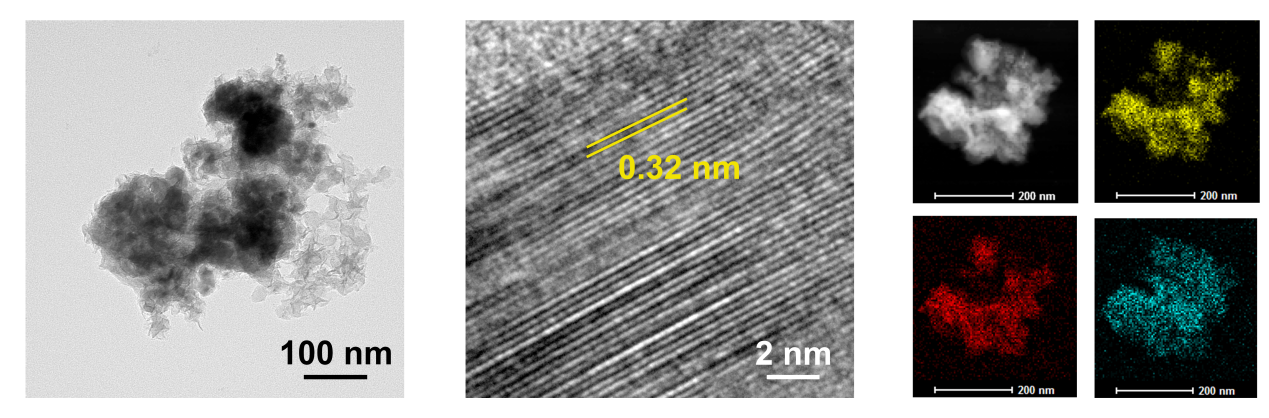


**Figure S5** TEM image (left), HRTEM image (center) and EDS energy spectrum (right) of CuS_0.4_Se_0.6_.


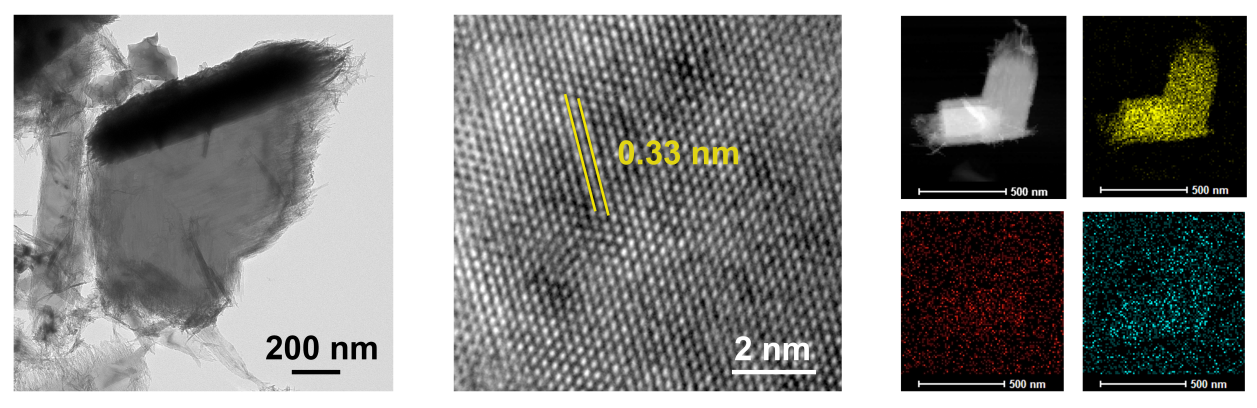


**Figure S6** TEM image (left), HRTEM image (center) and EDS energy spectrum (right) of CuSe.


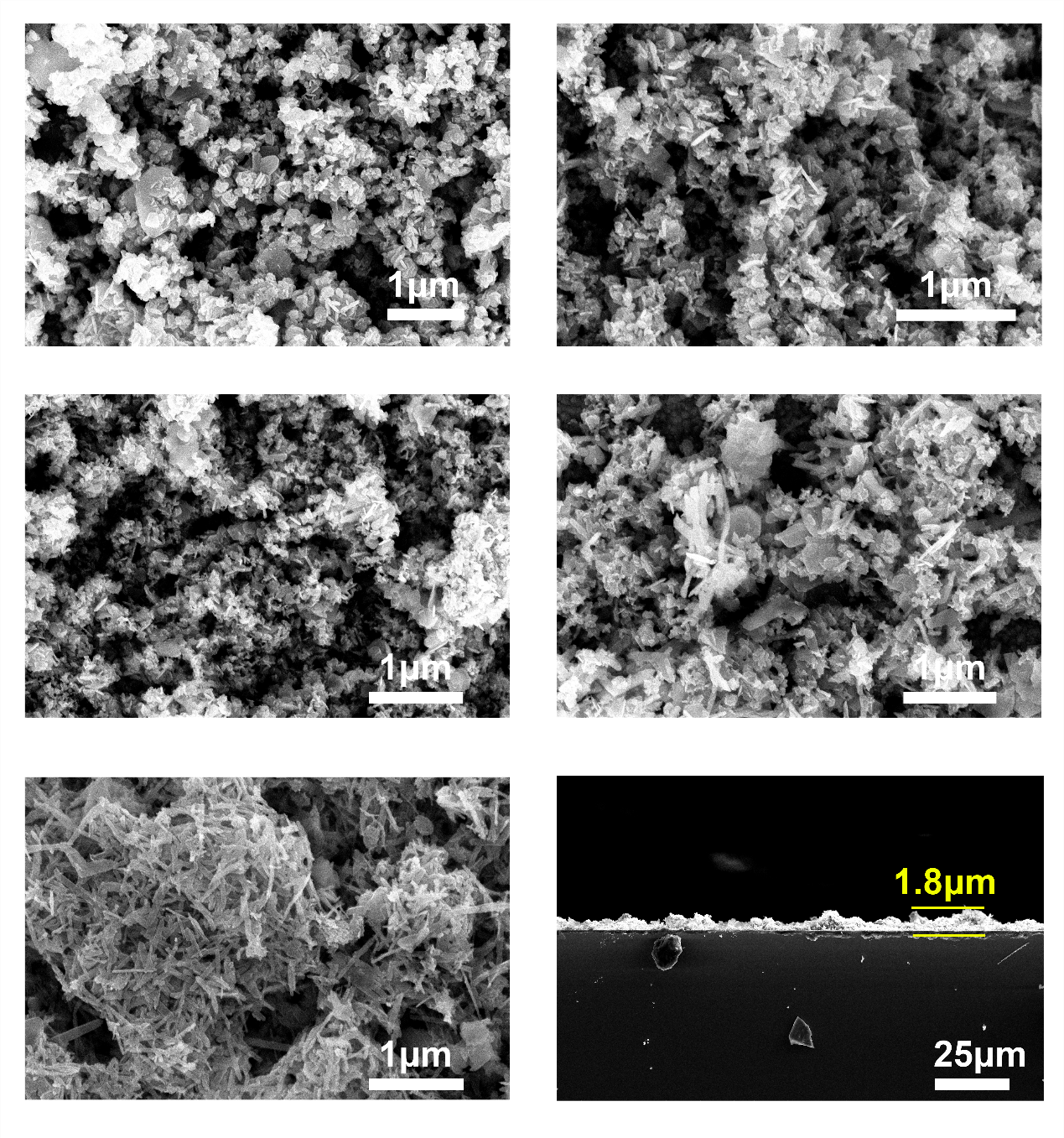


**Figure S7** SEM image of CuS thin film sprayed with an inkjet printer.


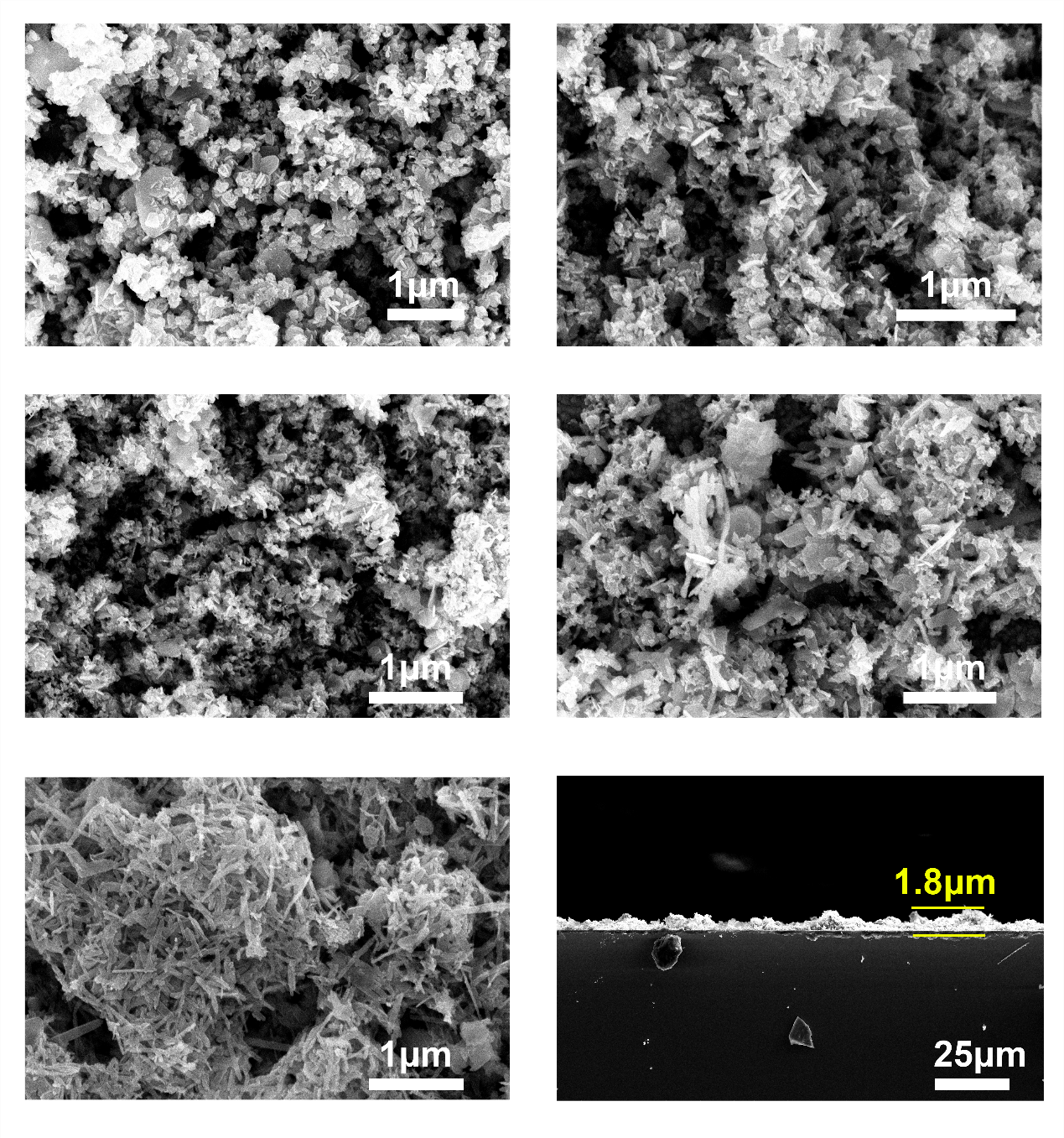


**Figure S8** SEM image of CuS_0.6_Se_0.4_ thin film prepared by spray coating.


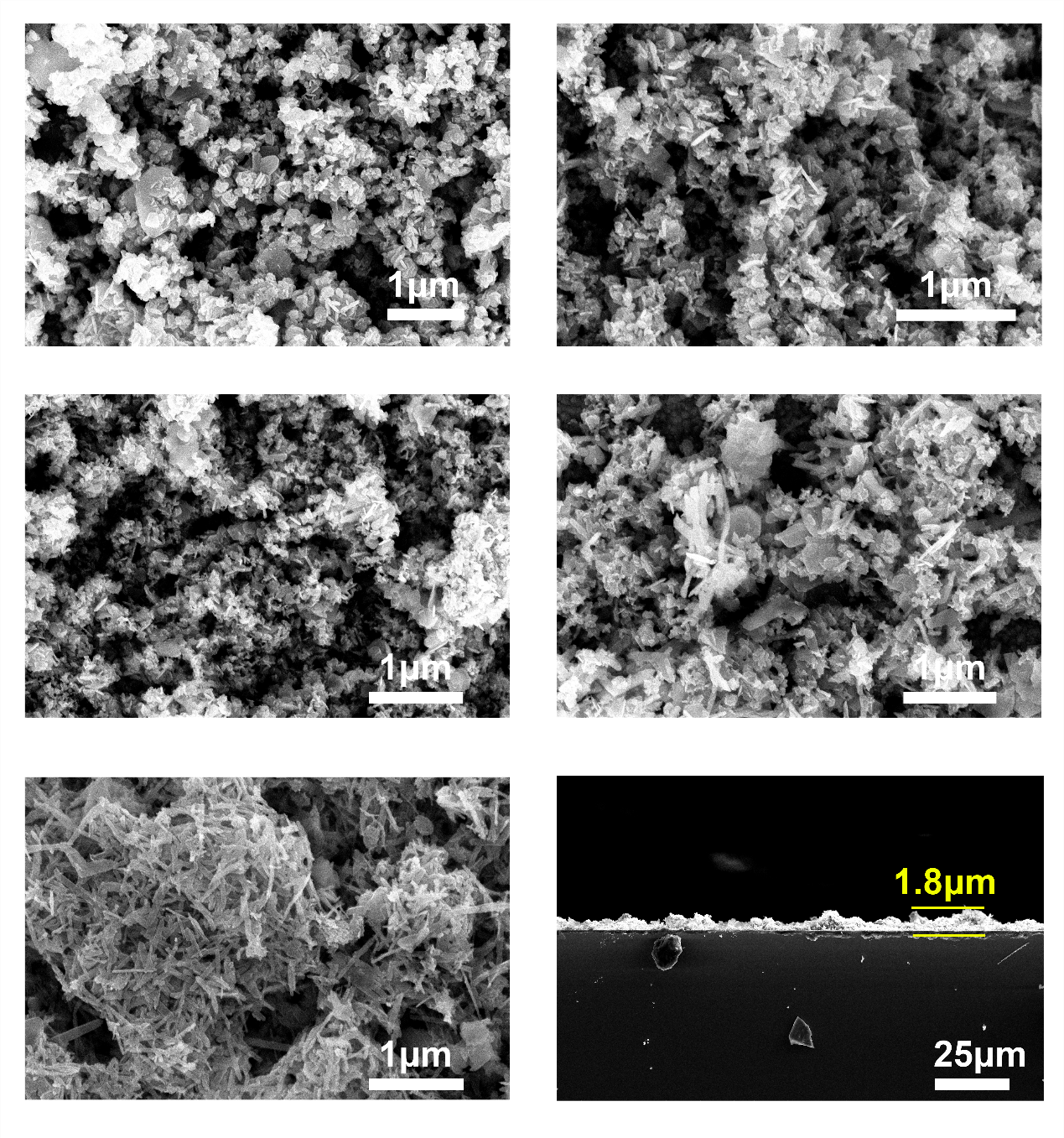


**Figure S9** SEM image of CuS_0.5_Se_0.5_ thin film prepared by spray coating.


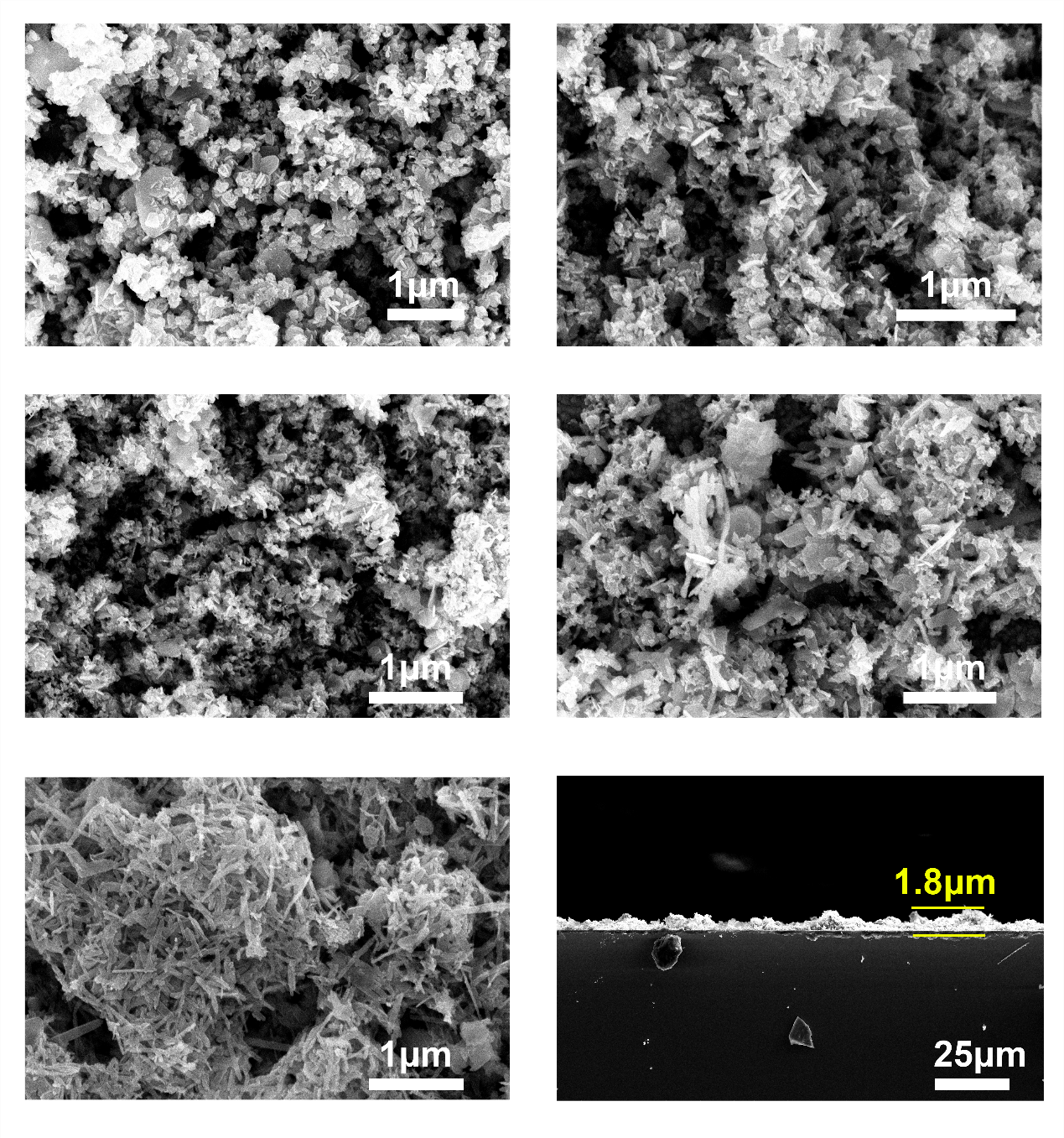


**Figure S10** SEM image of CuS_0.4_Se_0.6_ thin film prepared by spray coating.


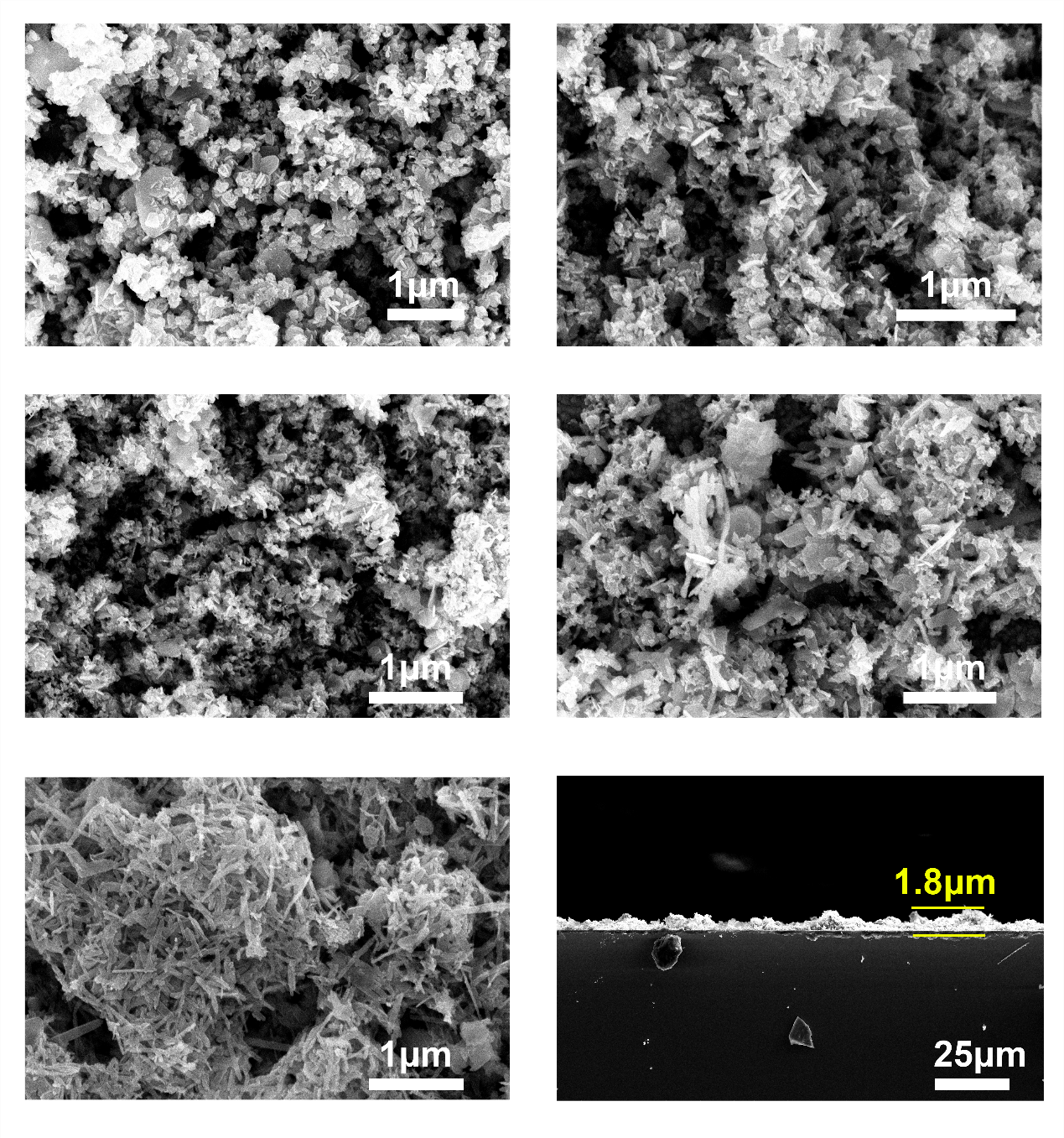


**Figure S11** SEM image of CuSe thin film prepared by spray coating.


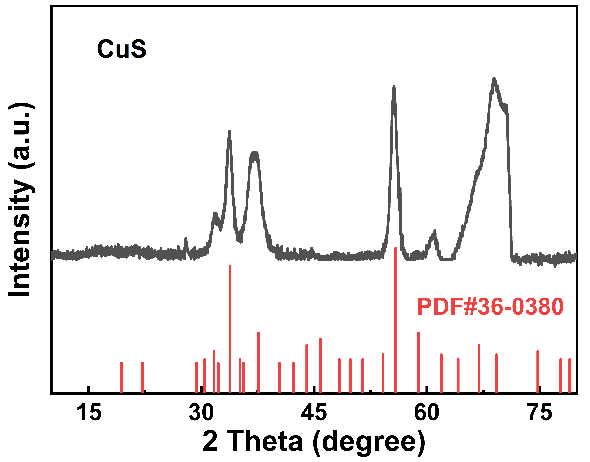


**Figure S12** XRD pattern of CuS, corresponding to standard card PDF#36-0380.


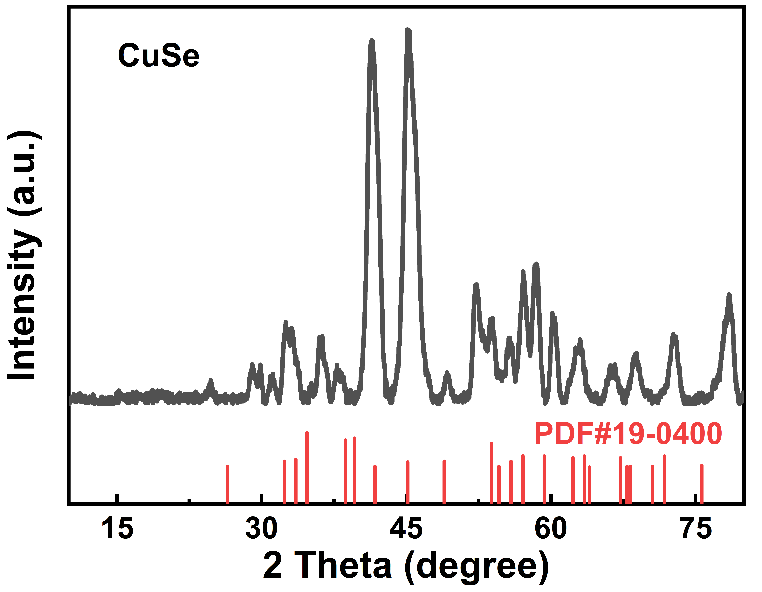


**Figure S13** XRD pattern of CuSe, corresponding to standard card PDF#19-0400.


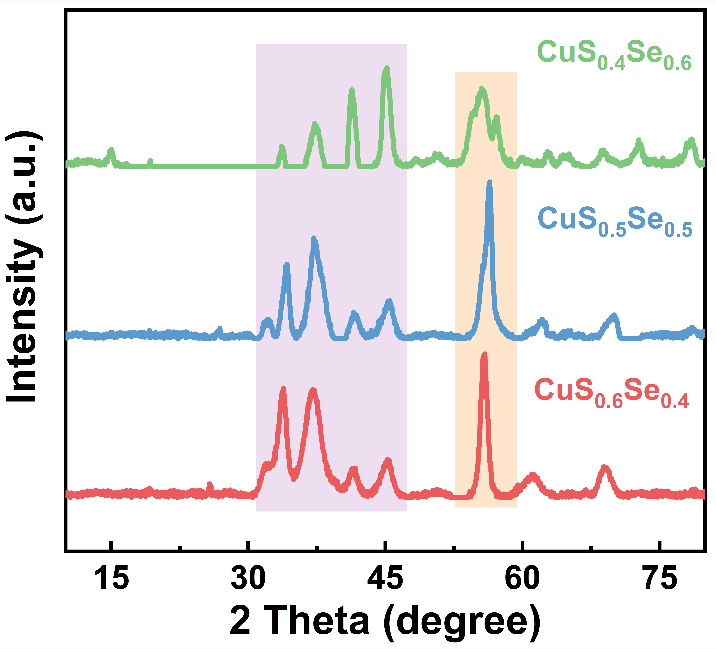


**Figure S14** XRD pattern of CuS0.6Se0.4、CuS0.5Se0.5 and CuS0.4Se0.6. The colored areas are labeled with their peak positions and intensity changes.


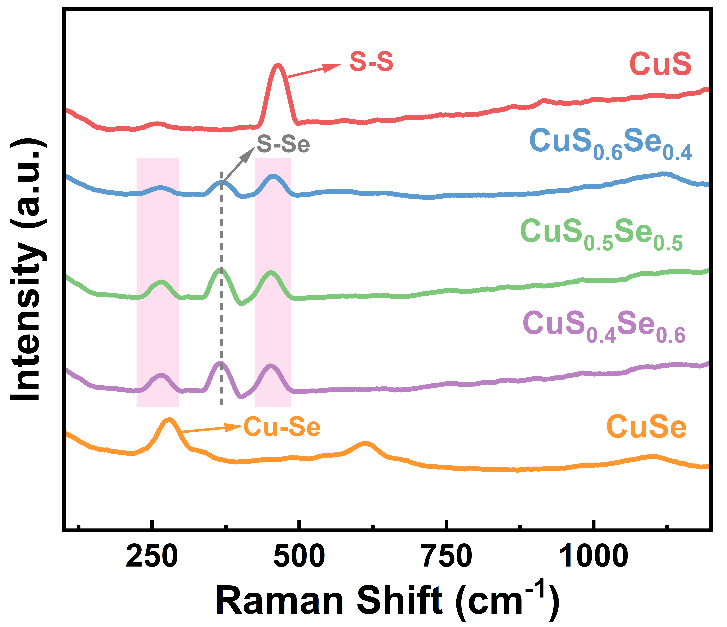


**Figure S15** Raman spectra of five groups of samples.


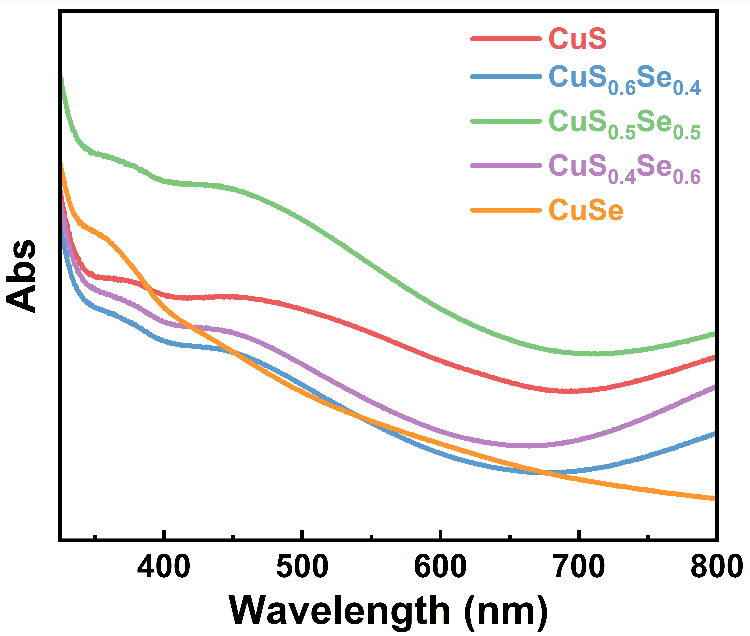


**Figure S16** UV-Vis absorption spectra of five groups of samples.


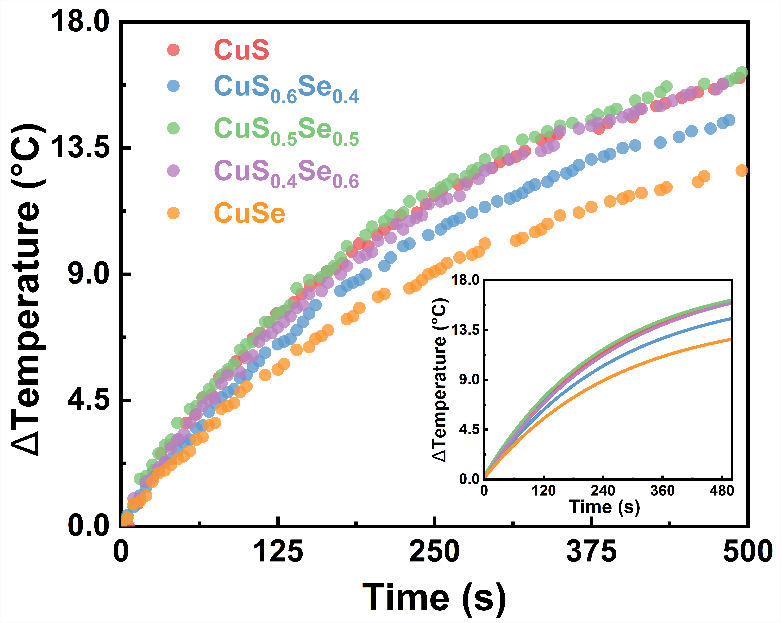


**Figure S17** Temperature variation of CSS under 365 nm illumination, inset shows the fitted curves.


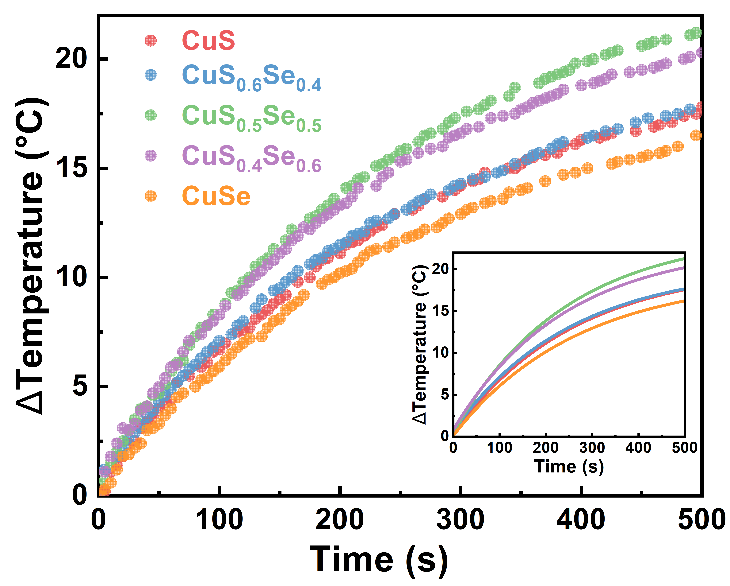


**Figure S18** Temperature variation of CSS under 405 nm illumination.


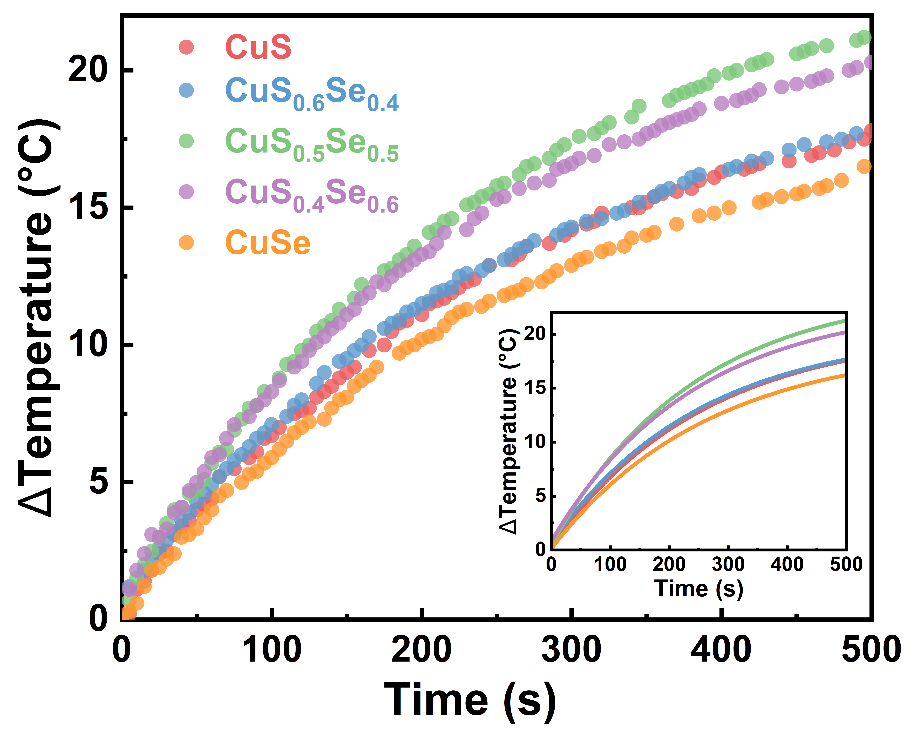


**Figure S19** Temperature variation of CSS under 450 nm illumination.


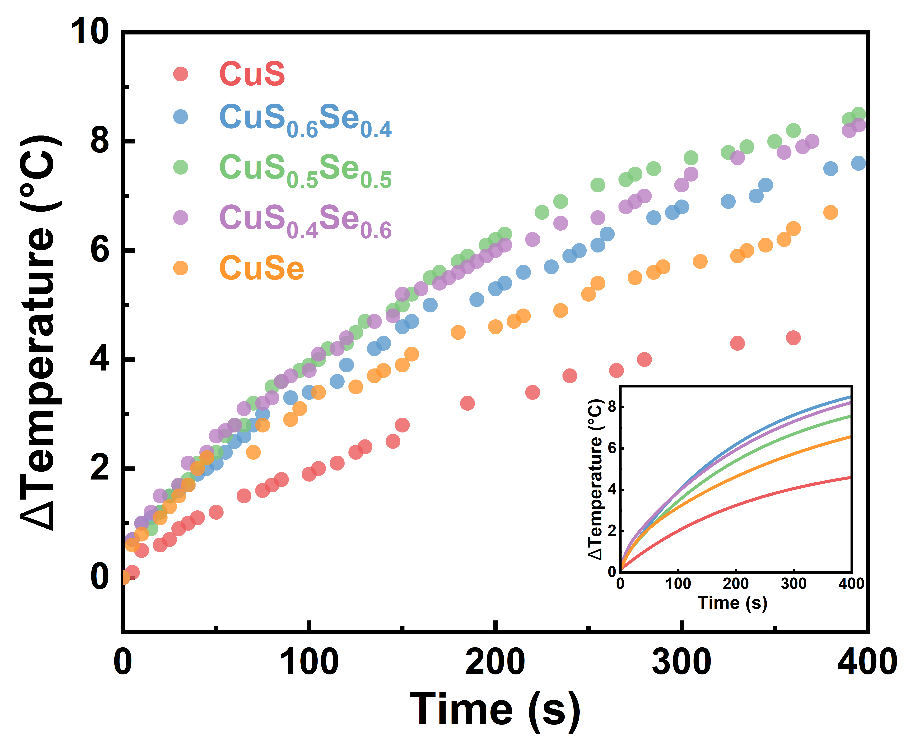


**Figure S20** Temperature variation of CSS under 515 nm illumination.


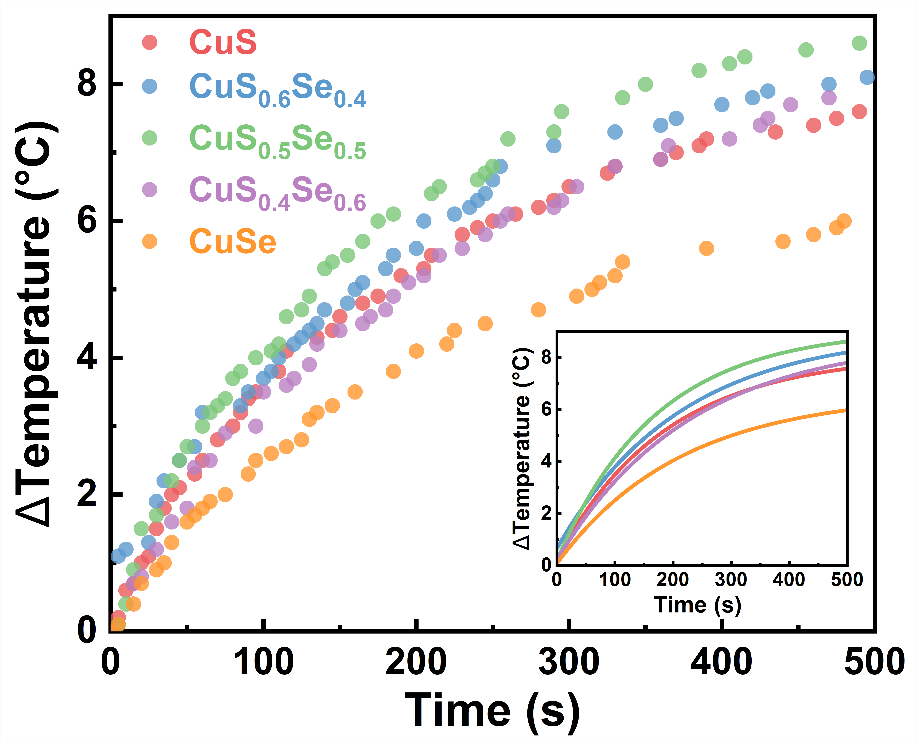


**Figure S21** Temperature variation of CSS under 808 nm illumination.

The selenide of copper sulfide was investigated for its optimal selenization ratio. As shown in **Fig. S2-S6**, we prepared five sets of CuS_y_Se_1-y_ with different ratios: y = 0, y = 0.4, y = 0.5, y = 0.6, and y = 1, by controlling the amount of selenium powder using a hydrothermal growth method. We observed that the crystal spacing increases with the selenization ratio, attributed to the larger size of selenium atoms compared to sulfur. The selenide ratio of the composite was also confirmed in the selenium composite. The microscopic morphology of the product changes significantly with increasing y values, with lamellae initially forming a hexagonal structure and subsequently growing in the (006) direction, leading to the development of blade-like lamellae, as depicted in **Fig. S7-S11**. And all of these lamellae are characterized by copper-sulfur compounds that are prone to clustering. The XRD test results show that when y = 1, as depicted in **Fig. S12**, the diffraction peaks of CuS closely match the standard peaks for the hexagonal phase of CuS, with lattice parameters of a = 3.792 Å and c = 16.344 Å. Conversely, when y = 0, CuSe exhibits better crystallinity (Fig. S13), with lattice parameters of a = b = 3.939 Å and c = 17.250 Å. The (006) plane shows stronger reflections compared to standard XRD patterns. **Fig. S14** illustrates that diffraction peaks of elemental Se appeared and increased in intensity with the selenization ratio, while the diffraction peaks of elemental S gradually diminished. Consequently, the diffraction peaks of the Cu (001) plane also decreased in intensity as a percentage of the total, due to the larger mass and volume of Se atoms relative to S atoms. The Raman spectroscopy results (**Fig. S15**) reveal distinct Raman vibrational peaks corresponding to S-S bonds in CuS. With increasing selenization, peaks associated with S-Se and Cu-Se bonds gradually emerge. Upon complete selenization, only a strong peak at 260 cm⁻¹ remains, which corresponds to the first longitudinal optical (LO) phonon mode of Cu-Se vibration. Additionally, the UV-Vis absorption and transmission spectra of the five sample sets (**Fig. S16**) exhibit notable absorption properties in the 360 - 480 nm range. The increasing proportion of selenization correlates with a rising bandgap, with y = 0.5 showing a pronounced dominant absorption feature. Measurement and comparison of temperature increases under light reveal that this sample set achieves the highest photothermal conversion temperature across different wavelengths of light, as illustrated in **Fig. S17-S21**. Therefore, y = 0.5, which is CuS0.5Se0.5, was selected for further study and will be referred to as CSS.


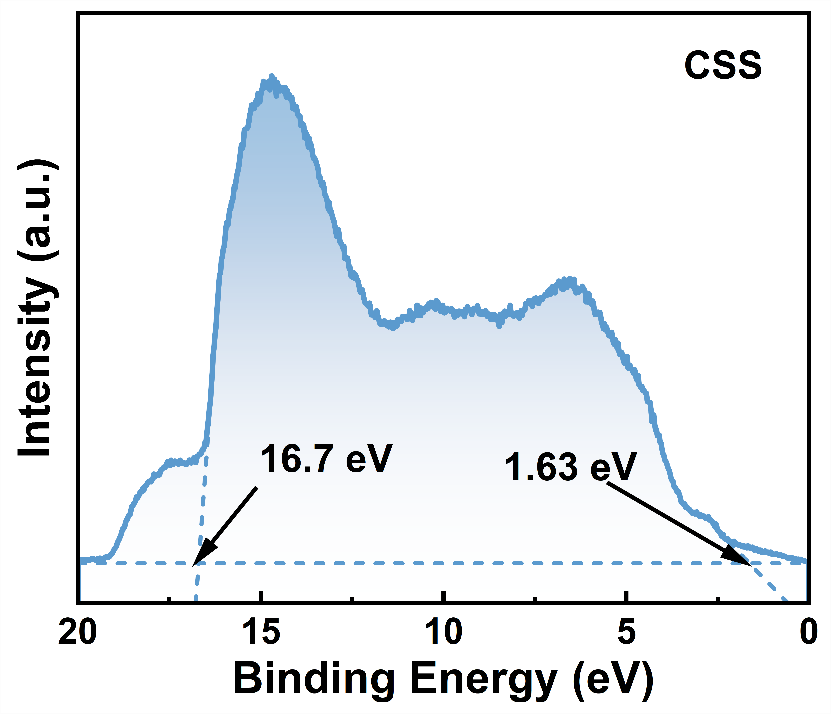


**Figure S22** UPS Spectrum for CSS.


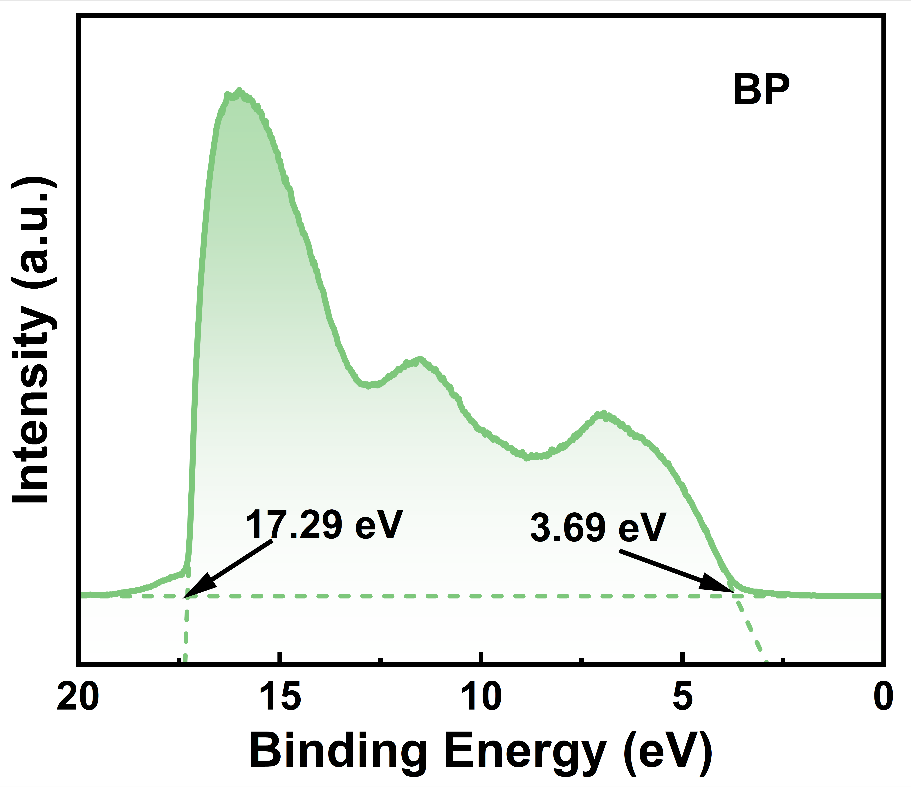


**Figure S23** UPS Spectrum for BP.


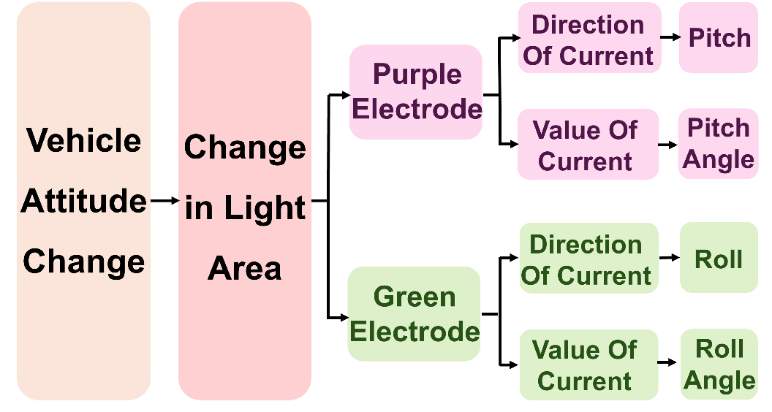


**Figure S24** The workflow of ARB.


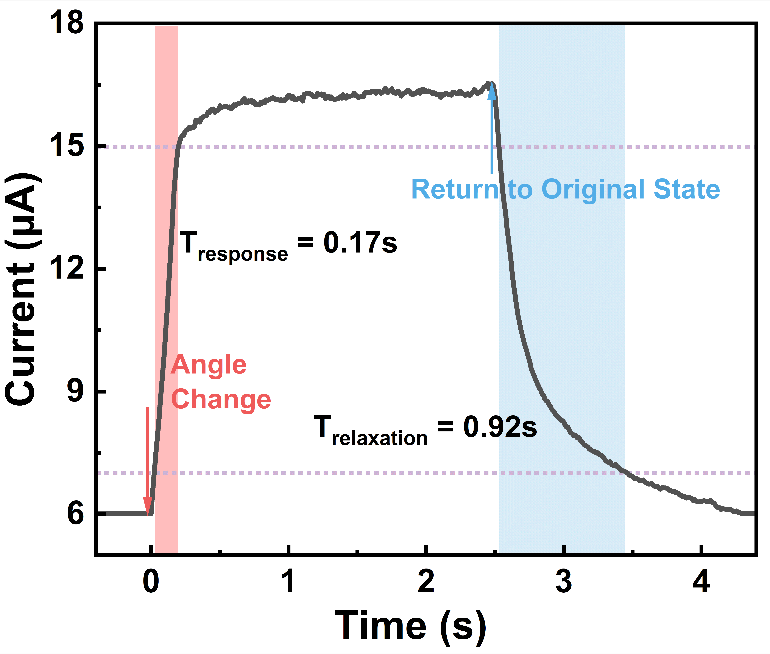


**Figure S25** The time-resolved response of the ARB to angle change with the red and blue zones corresponding to the response and relaxation time.

**Table S1** Comparison of photothermal properties of CSS@BP composites with other works.

| **Name** | **Photothermal Conversion Efficiency** | **∆Temperature** | **Reference** |
| --- | --- | --- | --- |
| **CSS@BP** | **68.9%** | **48.4°C** | **This Work** |
| **CuS (V_s_)** | **41.3%** | **29.8 °C** | **[1]** |
| **CuS-T150** | **55.41%** | **36 °C** | **[2]** |
| **CuS@Cu-MOF** | **39.6%** | **28.4 °C** | **[3]** |
| **CuS-C_2_** | **43.4%** | **48 °C** | **[4]** |
| **PCM@PUF-CuS@MSR PCC** | **51.25%** | **37 °C** | **[5]** |
| **BP** | **30.84%** | **21.3 °C** | **[6]** |
| **BPQD** | **28.9%** | **26 °C** | **[7]** |

**Table S2** Measured parameters of ARB at fuselage and wing in different environments under the same deflection state correspond to **Fig. 6f** (deflection angle is 30°).

| **Number** | **External**  **Conditions** | **Parameter** | **Actual value** | | **Measured Groups** |
| --- | --- | --- | --- | --- | --- |
|  |  |  | **Fuselage** | **Wing** |  |
| **1** | **Dark** | **-** | **29.83°~30.16°** | **29.82°~30.09°** | **16** |
| **2** | **Strong Light** | **Car headlamp, 60 W** | **29.85°~30.08°** | **29.84°~30.09°** | **16** |
| **3** | **Magnetic Field** | **0.5 T** | **29.82°~30.1°** | **29.80°~30.11°** | **16** |
| **4** | **Wind** | **50 km/h** | **29.82°~30.07°** | **29.84°~30.09°** | **16** |
| **5** | **Prolonged Repetition** | **Change angle every 1 min for 30 mins.** | **29.83°~30.10°** | **29.87°~30.12°** | **30** |

**Table S3** The performance comparison of ARB with other similar estimation techniques.

| **Number** | **Comments** | **Tolerance Range** | **Reference** |
| --- | --- | --- | --- |
| **1** | **Photothermoelectric Converter** | **＜ 0.2°** | **This work** |
| **2** | **Fuzzy Adaptive Attitude Estimation** | **0.2° ~ 2.6°** | **[8]** |
| **3** | **Considering Signal Measurement Characteristic** | **≈ 0.2°** | **[9]** |
| **4** | **Precision imu based estimation** | **0.5°** | **[10]** |
| **5** | **Deep Learning-Based Hybrid Approach** | **≥ 1°** | **[11]** |
| **6** | **Based on Low-Cost Sensors** | **≤ 0.5°** | **[12]** |

The equation for the relationship between the ARB current oscillator (C) and the angle of deflection (A) is:

$$A=13.72\left[ 1+exp\left( C-44.85 \right) \right]-0.00732$$

Reference

[1] S. Mo, Y. Song, M. Lin, J. Wang, Z. Zhang, J. Sun, D. Guo, L. Liu, *J. Colloid Interface Sci.*, **2022**, 608, 2896-2906.

[2] Z. Zhang, J. Wen, J. Zhang, D. Guo, Q. Zhang, *Adv. Healthc. Mat.*, **2023**, 12(1), 2201746.

[3] P. Geng, N. Yu, D.K. Macharia, R. Meng, P. Qiu, C. Tao, M. Li, H. Zhang, Z. Chen, W. Lian, *Chem. Eng. J.*, **2022**, 441, 135964.

[4] X. Wang, X. Sun, W. Liu, H. Li, J. Wang, D. Wang, *J Colloid Interface Sci.*, **2024**, 657, 142-154.

[5] W. Chen, X. Liang, W. Fu, S. Wang, X. Gao, Z. Zhang, Y. Fang**,** *ACS Appl. Energy Mater.*, **2022**, 5(7), 9109-9117.

[6] D. Xu, J. Liu, Y. Wang, Y. Jian, W. Wu, R. Lv**,** *ACS Biomater. Sci. Eng.*, **2020**, 6(9), 4940-4948.

[7] H. Ding, D. Wang, H. Huang, X. Chen, J. Wang, J. Sun, J. Zhang, Lu Lu, B. Miao, Y. Cai, K. Fan, Y. Lu, H. Dong, X. Yan, G. Nie, M. Liang, *Nano Res.*, **2022**, 15, 1554-1563.

[8] W. Youn, M.B. Rhudy, A. Cho, H. Myung, *IEEE SENS. J.*, 20.3, **2020**, 1456-1472.

[9] W. Liu, X. Xia, L. Xiong, Y. Lu, L. Gao, Z. Yu, *IEEE SENS. J.*, 21.19, **2021**, 21675-21687.

[10] M. Hrgetić, J. Deur, *2020 IEEE I2MTC*, **2020**, 1-6.

[11] K. Cho, H. Lee, *IEEE Access*, **2024**, 12, 157165-157178.

[12] X. Ding, Z. Wang, L. Zhang, *IEEE TII*, 2021, 18.7, 4466-4476.
